# Supplementary material for: Rebound effects undermine carbon footprint reduction potential of autonomous electric vehicles
Source: Nat Commun. 2023 Oct 6;14:6258. doi: 10.1038/s41467-023-41992-2 (PMC10558530; doi:10.1038/s41467-023-41992-2)
Supplement: Supplementary file 1 — Supplementary Information [file 41467_2023_41992_MOESM1_ESM.pdf]

Supplementary Information File

For

Rebound Effects Undermine Carbon Footprint Reduction Potential of Autonomous Electric Vehicles

Nuri C. Onat [1,*], Jafar Mandouri [1,2], Murat Kucukvar [3], Burak Sen [4], Saddam A. Abbasi [5,6], Wael Alhajyaseen [1,7], Adeeb A. Kutty [1], Rateb Jabbar[8], Marcello Contestabile[9,10], Abdelmagid Hamouda [11]

[1] Qatar Transportation and Traffic Safety Center, College of Engineering, Qatar University, Doha, Qatar;
[2] Engineering Management, College of Engineering, Qatar University, Doha, Qatar.
[3] Department of Business Ethics & Legal Studies, Daniels College of Business, University of Denver, Denver, Colorado, USA.
[4] SAU Center for Research & Development, and Applied Research (SARGEM), Faculty of Engineering, Sakarya University, 54050 Sakarya, Turkey.
[5] Statistics Program, Department of Mathematics, Statistics, and Physics, College of Arts and Sciences, Qatar University, 2713, Doha, Qatar.
[6] Statistical Consulting Unit, College of Arts and Sciences, Qatar University, Doha, Qatar
[7] Department of Civil and Environmental Engineering, College of Engineering, Qatar University, Doha, Qatar.
[8] The KINDI Center for Computing Research, College of Engineering, Qatar University, Doha, Qatar.
[9] Qatar Environment and Energy Research Institute, Hamad Bin Khalifa University, Qatar Foundation, Doha, Qatar.
[10] Imperial College London, Faculty of Natural Sciences, Centre for Environmental Policy, London, United Kingdom.
[11] Department of Mechanical and Industrial Engineering, College of Engineering, Qatar University, Doha, Qatar.
*Corresponding Author's email: onat@qu.edu.qa

TABLE OF CONTENTS

1. Autonomous Vehicles Supplementary Information 6
2. Survey Results 8
 2.1 Annual Travelling Distances Analysis 15
 2.2 Generalized Linear Models 26
 2.3 Autonomous Vehicles Adoption Perceptions 27
3. Life Cycle Assessment Supporting Information 28
 3.1 Manufacturing Phase 29
 3.2 Operation Phase 38
 3.3 End-of-Life Phase 39
 3.4 Monte Carlo Simulation 40

LIST OF FIGURES

Supplementary Figure 1 | Cluster bar charts for covariate demographic characteristics of respondents. 11
Supplementary Fig. 2 | Histogram of annual traveling distance. 15
Supplementary Fig. 3 | Histogram of rebound effect. 16
Supplementary Fig. 4| Minimum, average, and maximum annual rebound-adjusted traveling distances after bootstrapping. ... 18
Supplementary Fig. 5| Minimum annual traveling distance sample average histogram. 20
Supplementary Fig. 6 | Average annual traveling distance sample average histogram. 21
Supplementary Fig. 7 | Maximum annual traveling distance sample average histogram. 22
Supplementary Fig. 8 | Minimum rebound-adjusted annual traveling distance sample average histogram. 23
Supplementary Fig. 9 | Average rebound-adjusted annual traveling distance sample average histogram. 24
Supplementary Fig. 10 | Maximum rebound-adjusted annual traveling distance sample average histogram. 25
Supplementary Fig. 11 | Breakdown of life cycle emissions. 43

LIST OF TABLES

Supplementary Table 1 | Levels of Vehicle Automation 6
Supplementary Table 2 | Autonomous vehicle components 7
Supplementary Table 3 | Autonomous vehicles material composition 7
Supplementary Table 4 | Demographics of the respondents 8
Supplementary Table 5 Summary of annual traveling distances 19
Supplementary Table 6 | Generalized linear models results 27
Supplementary Table 7 | Autonomous vehicles proposed benefits summary 28
Supplementary Table 8 | Autonomous vehicles adoption concerns summary 28
Supplementary Table 9 | Autonomous vehicle components manufacturing checklist 31
Supplementary Table 10 | Autonomous vehicle components specifications & costs 32
Supplementary Table 11 | Autonomous vehicle components specifications & costs reference 33
Supplementary Table 12 | Monte Carlo simulation parameters for given scenarios 40
Supplementary Table 13 | Monte Carlo simulation results 41
Supplementary Table 14 | Manufacturing, operation, and end-of-life phases cost combinations summary 43

1. Abbreviations

AV	Autonomous Vehicle
ICV	Internal Combustion Engine
CO2	Carbon Dioxide
SAV	Shared Autonomous Vehicle
PV	Photovoltaics
LCA	Life Cycle Assessment
A-BEV	Autonomous Battery Electric Vehicle
CLD	Causal Loop Diagramming
SAEV	Shared Autonomous Electric Vehicle
MRIO	Multi-Region Input-Output
CH4	Methane
N2O	Nitrogen Dioxide
GWP	Global Warming Potential
LIB	Lithium-Ion Battery

1. Autonomous Vehicles Supplementary Information

According to the National Highway Traffic Safety Administration, there are six levels of vehicle automation as shown in Supplementary Table 1. Level 0 is the minimum level with no autonomy and involves full driver control. On the other hand, level 5 is the maximum level of autonomy, entails 100% autonomy and does not include any driver contribution. It is also referred to as a fully autonomous vehicle.

Supplementary Table 1 | Levels of Vehicle Automation (NHTSA) [1]. This table briefly summarizes the levels on autonomy and what tasks are automated within each autonomy level.

Level of vehicle automation	Description
Zero	The human driver does all the driving.
One	An advanced driver assistance system (ADAS) on the vehicle can sometimes assist the human driver with either steering or braking/accelerating, but not both simultaneously.
Two	An advanced driver assistance system (ADAS) on the vehicle can control both steering and braking/accelerating simultaneously under some circumstances. The human driver must continue to pay full attention (“always monitor the driving environment”) and perform the rest of the driving task.
Three	An automated driving system (ADS) on the vehicle can perform all aspects of the driving task under some circumstances. In those circumstances, the human driver must be ready to take back control at any time when the ADS requests the human driver to do so. In all other circumstances, the human driver performs the driving task.
Four	An automated driving system (ADS) on the vehicle can perform all driving tasks and monitor the driving environment to do all the driving – in certain circumstances. Humans need not pay attention in those circumstances.
Five	An automated driving system (ADS) on the vehicle can do all the driving in all circumstances. The human occupants are just passengers and need never be involved in driving.

Autonomous vehicles require four types of inputs for commuting: location, perception, prediction, and planning [2]. Starting with location, Sensors must be physically compatible with the vehicle's location on the map concerning nearby objects. For perception, Sensors must be able to detect a wide range of objects, including traffic signals and various forms and colors of signs. Other vehicles, people, animals, and lane markings are also revealed. For prediction, Algorithms for advanced engineering and machine learning systems examine all data to conclude what is the best decision to make in terms of accelerating, decelerating, or any other necessary action. Finally, for planning, the autonomous vehicle system must build on the inputs and utilize behavior prediction software to figure out

what the vehicle will do next by considering all the previously listed sorts of data. Autonomous vehicles require distinct components to commute, those components are shown in Supplementary Supplementary Table ***2***.

Supplementary Table 2 | Autonomous vehicle components. This table summarizes components within autonomous system and what purpose each components takes within the automation of driving.

Autonomous system component	Description
Light Detection and Ranging (LIDAR)	estimates the distance between the sensor and other objects, and it employs light beams (millions of laser pulses per second). The time it takes for light to reflect off a surface and return is measured by LIDAR. Based on the distance they can measure, there are three varieties of LIDAR for autonomous vehicles: short, mid, and long-range depending on how far objects can be detected
Ultrasonic Sensors	Ultrasonic sensors fitted on the vehicle offer information about items in the immediate vicinity. This sort of information is widely used in parking assistance, backup alarms, and blind-spot detection systems
Infrared Sensors	Infrared sensors detect lane lines, people, bicycles, and other objects that other sensors may struggle to detect in low light (at night) or specific scenarios (weather conditions such as rain)
Cameras	The vehicle's cameras detect both moving and stationary objects
Radio Detection and Ranging (RADAR)	A sensor that measures the distance between obstacles and the sensor using radio waves.
Software and Central Processing Unit (CPU)	Deep learning, sensor fusion, and surround vision are all combined in software & CPU. Other integrated functions include:<br><br>A- Global Positioning System (GPS): GPS triangulates a vehicle's location using satellites. One useful definition is Inertial Navigation Systems (INS), which use gyroscopes and accelerometers to determine a vehicle's location, orientation, and velocity. INS and GPS are frequently combined to increase accuracy.<br><br>B- Prebuilt Maps: When utilizing GPS and INS, these are utilized to correct location mistakes.<br><br>C- Dedicated Short-Range Communication (DSRC): a wireless communication system that allows automobiles to connect and other road users [3].<br><br>D- Vehicle to Everything (V2X) It also aids drivers in recognizing possible risks ahead, even if their vision is impaired [4].
Adaptive Cruise Control (ACC)	a system that is controlled by the autonomous vehicle system to automatically adjust the vehicle's speed based on the speed of the vehicle in front of it.

[5–8]

Supplementary Table 3 | Autonomous vehicles' material composition (Per vehicle). This table summarizes the material composition for each vehicle brand considered in this study.

Reference name	Iron ores (Kg)	Copper ores (Kg)	Nickel Ores (Kg)	Aluminum ores (Kg)	Glass (Kg)	Plastic (Kg)	Cobalt (Kg)	Manganese (Kg)	Magnesium (Kg)
China	372	129	56	393	49	653	19	19	6

Japan 1	501	173	71	525	65	766	24	24	8
Japan 2	384	135	63	409	51	587	21	21	6
India	350	117	37	356	45	535	12	12	6
Korea	500	169	60	514	65	765	20	20	8
US 1	528	188	94	568	70	807	31	31	8
US 2	480	169	80	512	63	734	27	27	8
Mexico	171	59	25	180	23	262	8	8	3
Germany 1	491	166	58	504	63	752	19	19	8
Germany 2	531	185	82	561	70	812	27	27	9
Turkey	426	155	87	467	57	650	29	29	7
France	394	136	57	414	52	603	19	19	6

2. Survey Results

Supplementary Table 4 | Demographics of the respondents. This table shows the demographic characteristic, and categories, frequency and percentage of each category in the sample n=330 samples

Demographic Characteristic	Category	Frequency	Percentage (%)
Age group	18 to 19	9	2.7
	20 to 24	55	16.7
	25 to 29	38	11.5
	30 to 34	44	13.3
	35 to 39	48	14.5
	40 to 44	49	14.8
	45 to 49	35	10.6
	50 to 54	33	10.0
	55 to 59	15	4.5
	60+	4	1.2
Marital status	Single	108	32.7
	Married	85	25.8
	Married with children	137	41.5
Employment	Full-time employee	261	79.1
	Part-time employee	22	6.7
	Student	39	11.8
	other	8	2.4
Education level	High school	26	7.9
	Diploma	27	8.2
	Bachelor's	178	53.9
	Graduate (Master's/PhD)	99	30.0
	Less than 20,000	206	62.4

	20,000 to 40,000	81	24.5
Income level (QAR)	Over 40,000	43	13.0
Individuals of 18 years or older in household	1 to 2	154	46.7
	3 to 4	91	27.6
	5 and over	85	25.8
Cars in Household	0	14	4.2
	1 to 2	195	59.1
	3 to 4	83	25.2
	Over 4	38	11.5
Driving experience (years)	0	12	3.6
	1 to 2	15	4.5
	3 to 5	38	11.5
	6 to 10	77	23.3
	Over 10	188	57.0
Vehicle’s ownership	Yes	290	87.9
	No	40	12.1

(a)

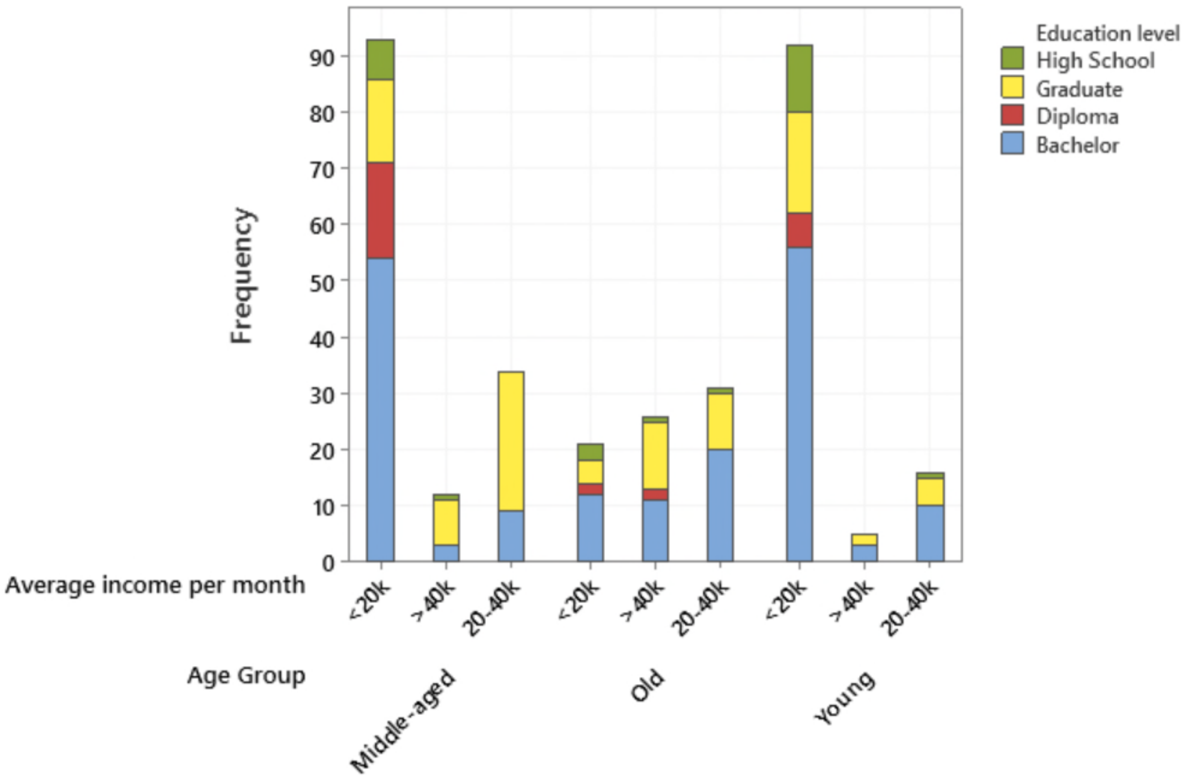
Frequency
Education level
High School
Graduate
Diploma
Bachelor
Average income per month
<20k
>40k
20-40k
<20k
>40k
20-40k
<20k
>40k
20-40k
Age Group
Middle-aged
Old
Young

(b)

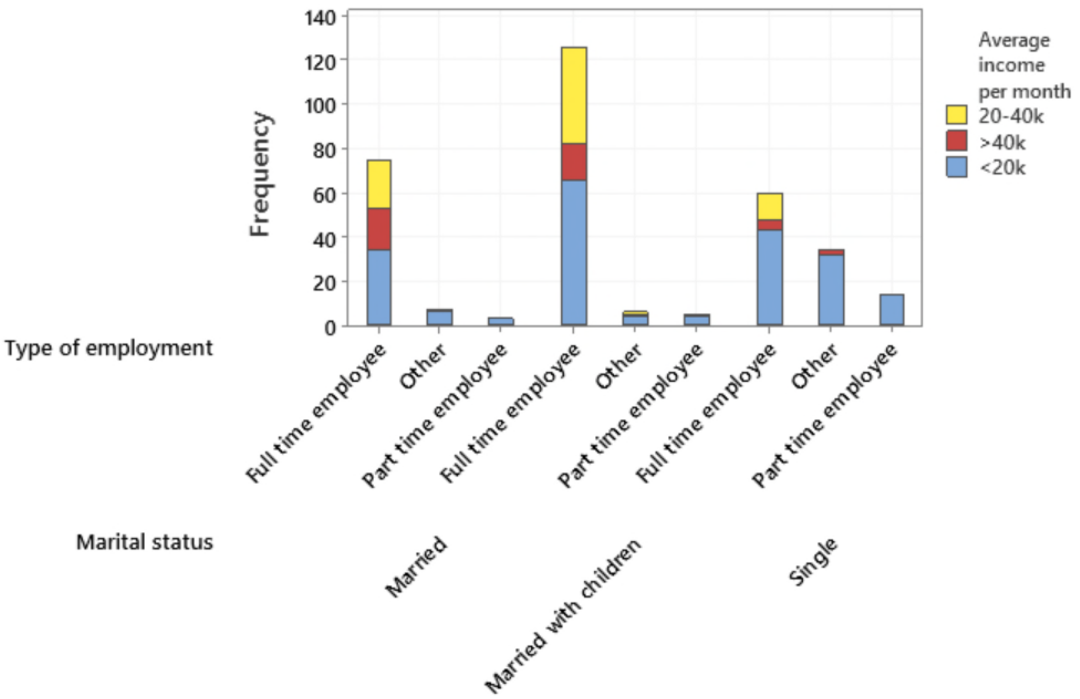

Supplementary Figure 1 | Cluster bar charts for covariate demographic characteristics of respondents. a, Cluster bar chart for age, education and average income of respondents. **b**, Cluster bar chart for marital status, type of employment and average income of respondents. This figure examines covariance between the different demographics among the respondents.

The following list provides the questions asked to the respondents and the choices whether they were provided with each question:

Section 1: Demographics

1. Age?

2. Marital status ? (Married with children, Married or Single)

3. Type of employment? (Full-time employee, Part-time employee, Student, Unemployed, Housewife, or Other)

4. Highest education level? (High school or less, Diploma degree, Bachelor's degree or Graduate (Masters or PhD))

5. Average income per month? (< 5,000 QR, 5,000-10,000 QR, 10,000-20,000 QR, 20,000-40,000 QR, or More than 40,000 QR)

6. Number of individuals (including yourself) above 18 years old in your household?

7. Total number of cars in your household?

Section 2: Commuting behavior

1. Number of years of driving experience?

2. Do you own a car? (Yes or no)

3. Average traveled distance per year? (<10,000 KM, 10,000- 20,000 KM, 21,000- 30,000 KM, 31,000- 40,000 KM, or >40,000 KM)

4. How Much you are willing to pay to buy a new car? (20,000- 40,000 QR, 40,000- 80,000 QR, 80,000- 160,000QR, 160,000-320,000QR or more than 320,000 QR)

5. For what purpose do you travel most by car (work, shopping, social, escort)?

6. On average how many kilometers do you travel per work trip? (5, 10, 15, 20, 25, 30, or not applicable)

7. On average what is the total travel time per day that you commute from home to work (only answer if you use a private car otherwise skip this question)? (Less than 10 min, 10-20min, 21-30min, 31-40min, or more than 40min)

8. What mode of travel do you mainly use for daily commute? (Bicycle, Bus, Metro, Private car (Driver), Private car (Passenger), Taxi, or walk)

Section 3: Autonomous vehicles

1. How much did you know about AV? (First time I heard about it, A simple background from (social media, newspaper, or the internet), A good background (knowing some of its properties), or a strong background (knowing what kind of technology is used in AV))

2. Select one statement representing your perception of the safety of AV:

A- Generally, AVs are safe, but I have a minor concern that something could go wrong

B- I am opposed to using AV unless I can override the control manually

C- I have no concerns about AV safety

D- I need to know a lot about AVs and their safety perforce

E- I think AVs are not safe and should not be allowed

2. Introducing AV will reduce the congestion on the roadways. (Strongly disagree, Disagree, Neutral, Agree or strongly disagree)

3. Introducing AV will reduce fuel consumption. (Strongly disagree, Disagree, Neutral, Agree or strongly disagree)

4. Introducing AV will reduce travel time. (Strongly disagree, Disagree, Neutral, Agree or strongly disagree)

5. Introducing AV will reduce parking costs. (Strongly disagree, Disagree, Neutral, Agree or strongly disagree)

6. AV can encourage me to travel on long-distance trips more often. (Strongly disagree, Disagree, Neutral, Agree or strongly disagree)

7. AV can allow me to visit places that I find difficult to reach through a regular car. (Strongly disagree, Disagree, Neutral, Agree or strongly disagree)

8. AV can make my travel more comfortable. (Strongly disagree, Disagree, Neutral, Agree or strongly disagree)

9. AV can eliminate human errors causing vehicle accidents. (Strongly disagree, Disagree, Neutral, Agree or strongly disagree)

10. I have concerns about securing the autonomous driving system from computer hackers. (Strongly disagree, Disagree, Neutral, Agree or strongly disagree)

11. I have concerns about the possibility of accidents between regular cars and autonomous vehicles. (Strongly disagree, Disagree, Neutral, Agree or strongly disagree)

12. I have concerns about the increase in maintenance cost in terms of (updating the computer system of the AVs and changing equipment’s costs). (Strongly disagree, Disagree, Neutral, Agree or strongly disagree)

13. I have concerns about the performance of autonomous vehicles in harsh environmental conditions (such as during rainy weather conditions) (Strongly disagree, Disagree, Neutral, Agree or strongly disagree)

14. Would you switch to using an autonomous vehicle if both regular and autonomous vehicles have the same travel time and travel cost? (Yes or no)

15. If you don’t own a driver's license yet, or not able to drive (elderly/person with a disability), or currently use another mode choice such as (public transport or taxi) would you switch to use autonomous vehicles? (Yes or no)

16. If you own an autonomous vehicle, how would your travel distance change have compared to your current travel patterns? (No change, Slight increase (10-20% more), Slight increase in distance (10-20% more), Moderate increase (20-50% more in distance), Moderate increase in distance (20-50% more), Considerable increase (50%-100%), Considerable increase in distance (50%-100%), Significant increase (at least 2 times more), Significant increase in distance (at least 2 times more))

2.1 Annual Travelling Distances Analysis

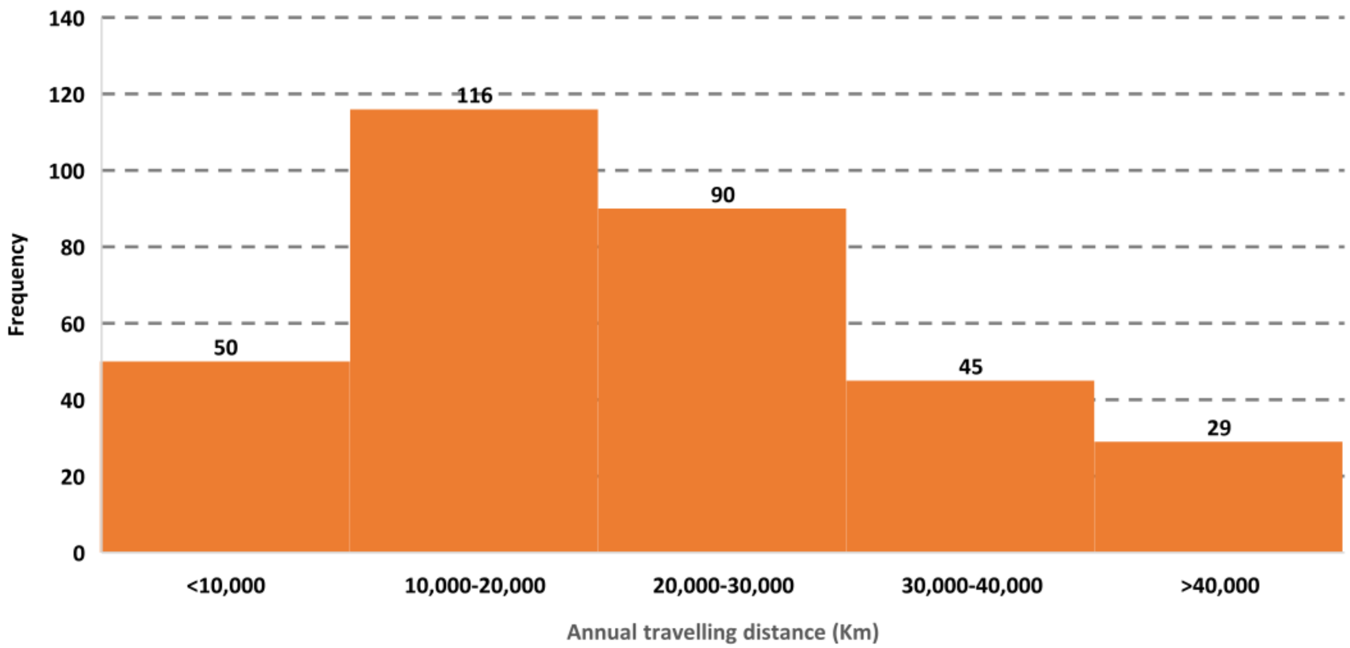

Supplementary Fig. 2 | Histogram of annual traveling distance. n=330 samples. This figure highlights how the respondents are distributed among annual traveling distance categories.

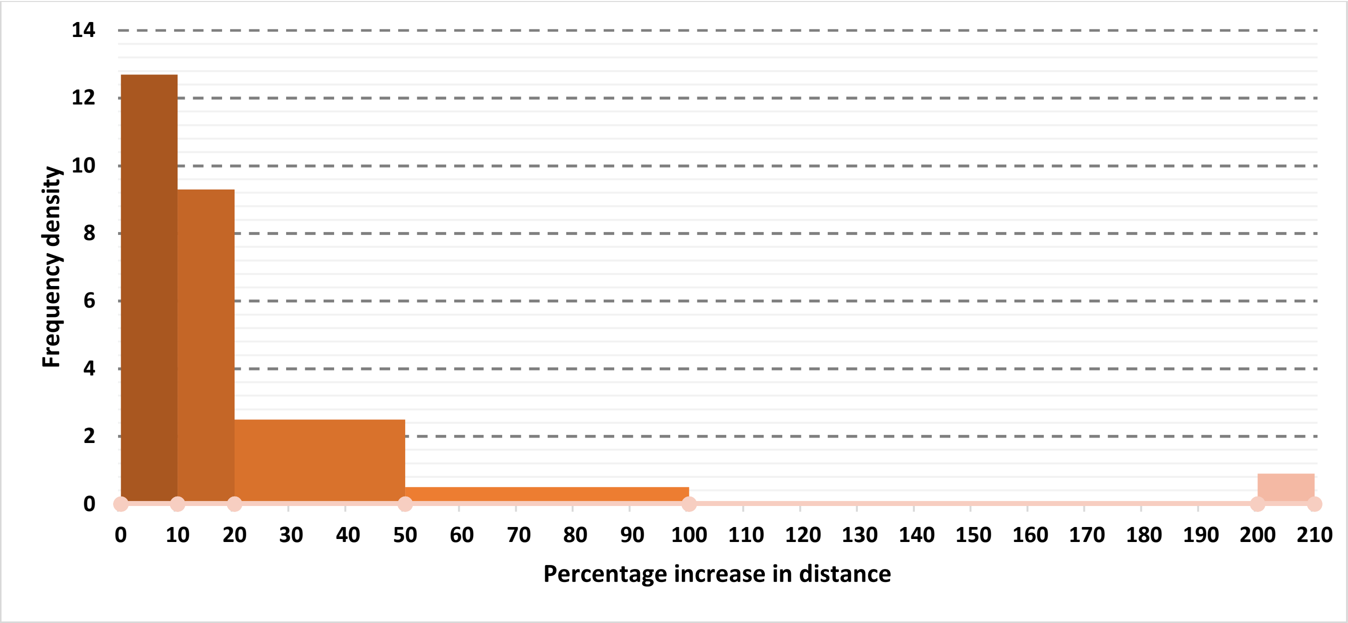

Supplementary Fig. 3 | Histogram of rebound effect. n=330 samples. This figure highlights how the respondents are distributed among rebound effect categories.

The annual rebound-adjusted traveling distance is calculated on three levels as follows:

1- Minimum rebound-adjusted annual traveling distance (MiRAATD): derived from multiplying the lower bound (LB) of the annual traveling distance and lower bound of rebound effect as follows in equation (1):

$$MiRAATD = (ATD)LB \times (RE)LB \quad (1)$$

Where ATD: Annual traveling distance; RE: Rebound effect

2- Average rebound-adjusted annual traveling distance (ARAATD): obtained using average annual traveling distance and average rebound effect in $ARAATD = (ATD)Avg \times (RE)Avg \quad (2)$).

$$\mathrm{ARAATD} = (\mathrm{ATD})\mathrm{Avg} \times (\mathrm{RE})\mathrm{Avg} \quad (2)$$

3- Maximum rebound-adjusted annual traveling distance (MRAATD): results of multiplication of the Upper bound (UB) of the Annual traveling distance and the Upper bound of the rebound effect as highlighted in equation (3).

$$\mathrm{MRAATD} = (\mathrm{ATD})\mathrm{UB} \times (\mathrm{RE})\mathrm{UB} \quad (3)$$

Due to the limited amount of data points of 330, Bootstrapping was done on the three sets of minimum, average, and maximum rebound-adjusted annual traveling distances by using sampling with replacement. Bootstrapping involved resampling a single dataset to produce a massive number of simulated samples. For each of the three sets, 330 values of rebound-adjusted annual traveling distances are sampled with replacement from the original rebound-adjusted annual traveling distance sets of 330 values each. The generated 330 values form one sample. Then, the average of the generated sample was calculated. This procedure was repeated to generate 10,000 sample averages using Python. **Error! Reference source not found.** Supplementary Supplementary Fig. ***4*** summarizes our bootstrapping results shown within a box and whisker plot. These three sets are of vital importance due to their use in estimating the rebound effect as well as estimating service life for autonomous vehicles. Furthermore, the bootstrapping procedure was also done on the original minimum, average, and maximum annual traveling distances to estimate the service life of battery electric vehicles. Then, the difference in the service life between autonomous and non-autonomous vehicles is used to estimate a percentage decrease in service life due to the rebound effect. This percentage represents the rebound effect which is calculated as in $RE= \left(\frac{MVM}{GATDSA}\right) - \ MVMGRAATDSAMVMGATDSA$

 (4).

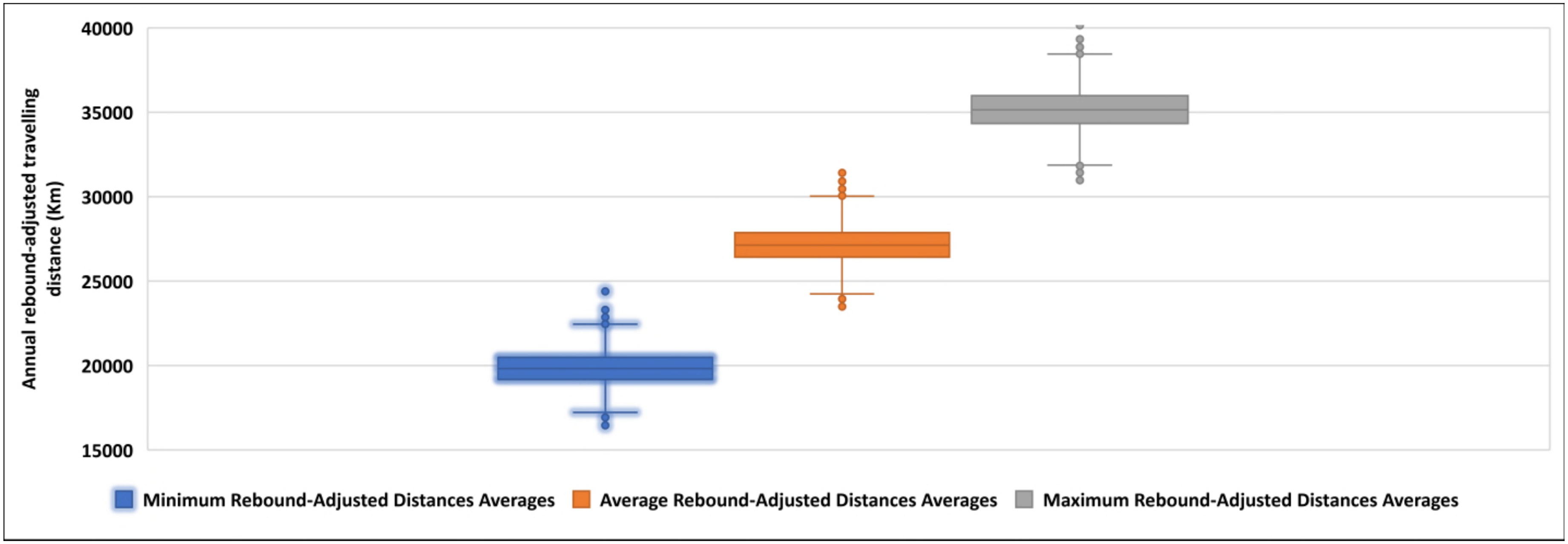

Supplementary Fig. 4| Minimum, average, and maximum annual rebound-adjusted traveling distances after bootstrapping. Box plots descriptive statistics are termed as follows: Minimum→ Min, Maximum→ Max, 1st quartile→1Q, 3rd quartile→ 3Q, Interquartile range→ IQR, Lower whisker→ LW, Upper whisker→ UW. For Minimum rebound-adjusted distances averages: Min=16459, Max= 24386, 1Q= 19179, 3Q= 20486, IQR= 1307, LW= 17219, UW= 22447. For Average rebound-adjusted distances averages: Min= 23494, Max= 31690, 1Q= 26416, 3Q= 27866, IQR= 1449, LW= 24243, UW= 30039. For Maximum rebound-adjusted distances averages: Min= 30967, Max= 40605, 1Q= 34334, 3Q= 35982, IQR= 1647, LW= 31863, UW= 38453

$$RE = \frac{\left(\left(\frac{MVM}{GATDSA}\right) - \left(\frac{MVM}{GRAATDSA}\right)\right)}{\left(\frac{MVM}{GATDSA}\right)} \qquad (4)$$

Where RE: Rebound effect; MVM: maximum vehicle milage (assumed to be 300,000 Km); GATDSA: generated annual traveling distances samples average; GRAATDSA: generated rebound-adjusted annual traveling distances samples average.

The following table summarizes the annual traveling distance sets. The minimum, average, and maximum annual traveling distances are extracted from the survey, where each reading reflects the participant's annual traveling distance values. As for minimum, average, and maximum annual traveling distance sample averages, they were extracted from carrying the bootstrapping simulation on the three original distances sets extracted from the survey. Lastly for minimum, average, and maximum rebound-adjusted annual traveling distance sample averages, we took the three original distances set, adjusted them with the rebound effect to reflect the increased annual travel due to autonomy, and then applied bootstrapping simulation.

Supplementary Table 5 Summary of annual traveling distances. This table demonstrates the findings of the traveling distances analysis for original 330 point data sets as well as 10,000 samples produced from bootstrapping.

Distance distribution	n	Mean	Standard deviation	Minimum	Median	Maximum
Minimum annual traveling distance	330	19,836	17,496	0	20,000	120,000
Average annual traveling distance	330	27,171	19,439	5,000	25,000	135,000
Maximum annual traveling distance	330	35,182	21,873	10,000	30,000	150,000
Minimum annual traveling distance sample average	10,000	16,570	644	14,134	16,565	18,936
Average annual traveling distance sample average	10,000	21,576	639	19,316	21,565	23,997
Maximum annual traveling distance sample average	10,000	26,574	640	24,407	26,565	29,027
Minimum rebound-adjusted annual traveling distance sample average	10,000	19,842	967	16,459	19,812	24,386
Average rebound-adjusted annual traveling distance sample average	10,000	27,158	1076	23,494	27,130	31,690
Maximum rebound-adjusted annual traveling distance sample average	10,000	35,170	1218	30,967	35,146	40,605

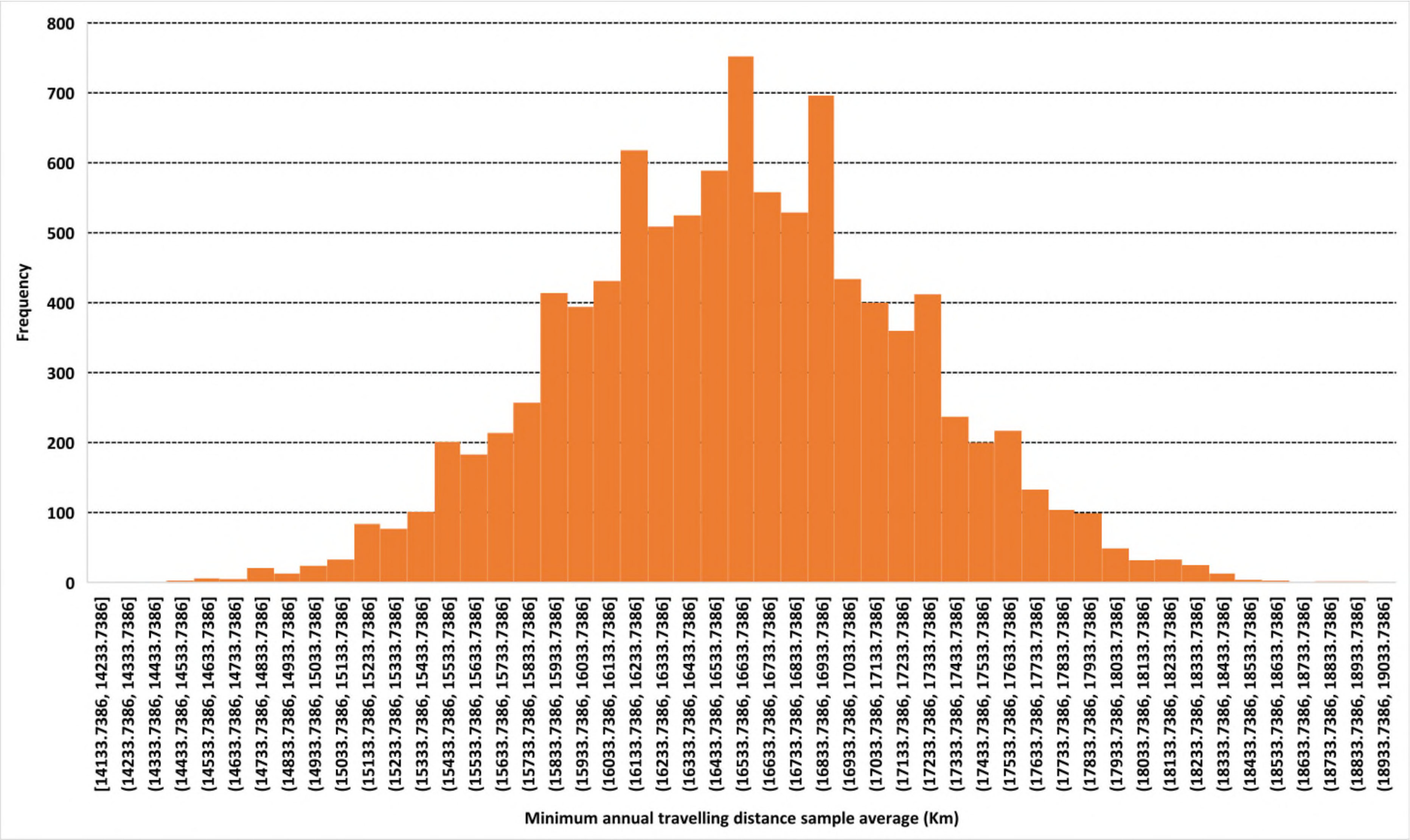

Supplementary Fig. 5| Minimum annual traveling distance sample average histogram. n=10,000 samples

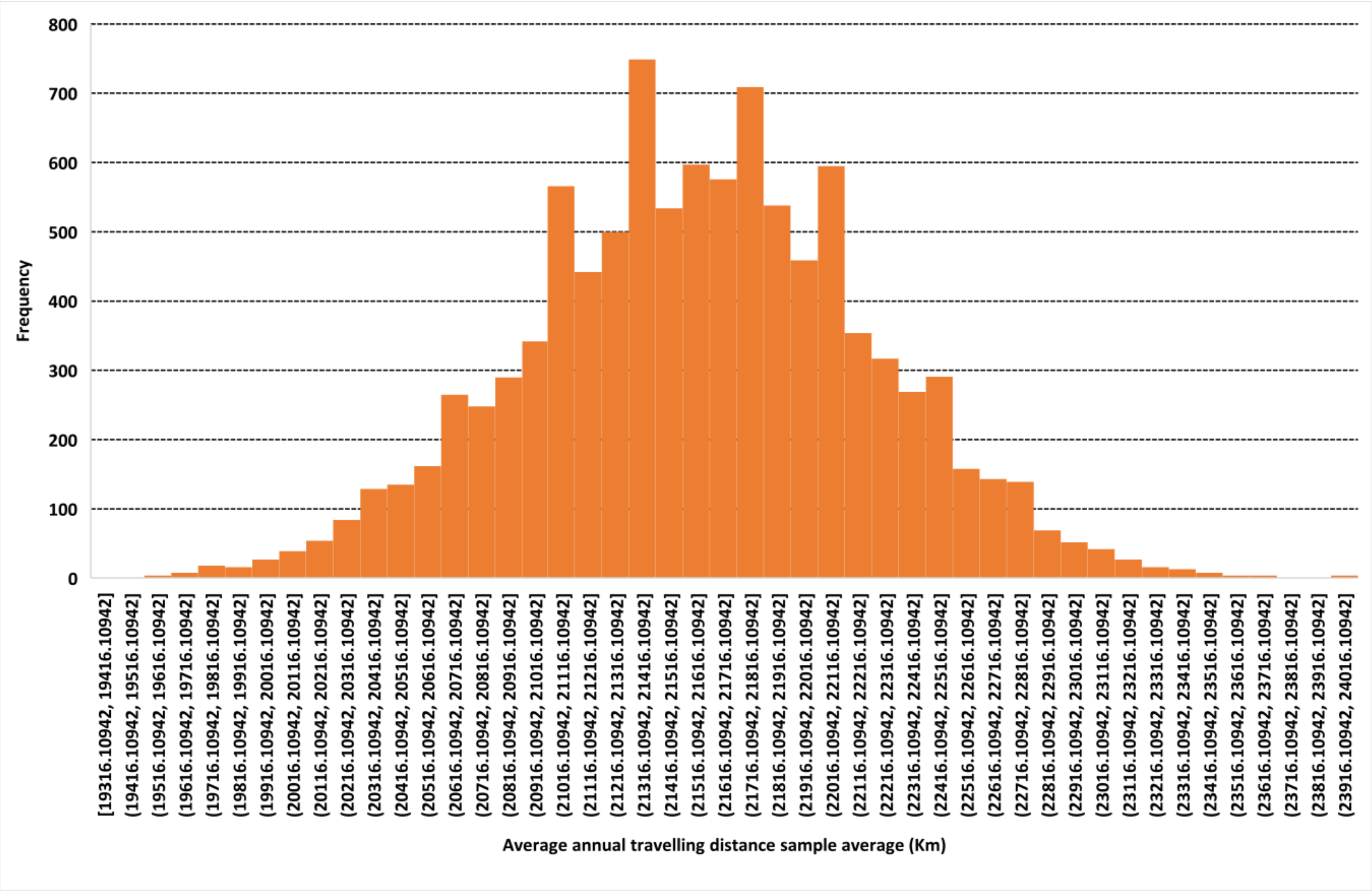

Supplementary Fig. 6 | Average annual traveling distance sample average histogram. n=10,000 samples

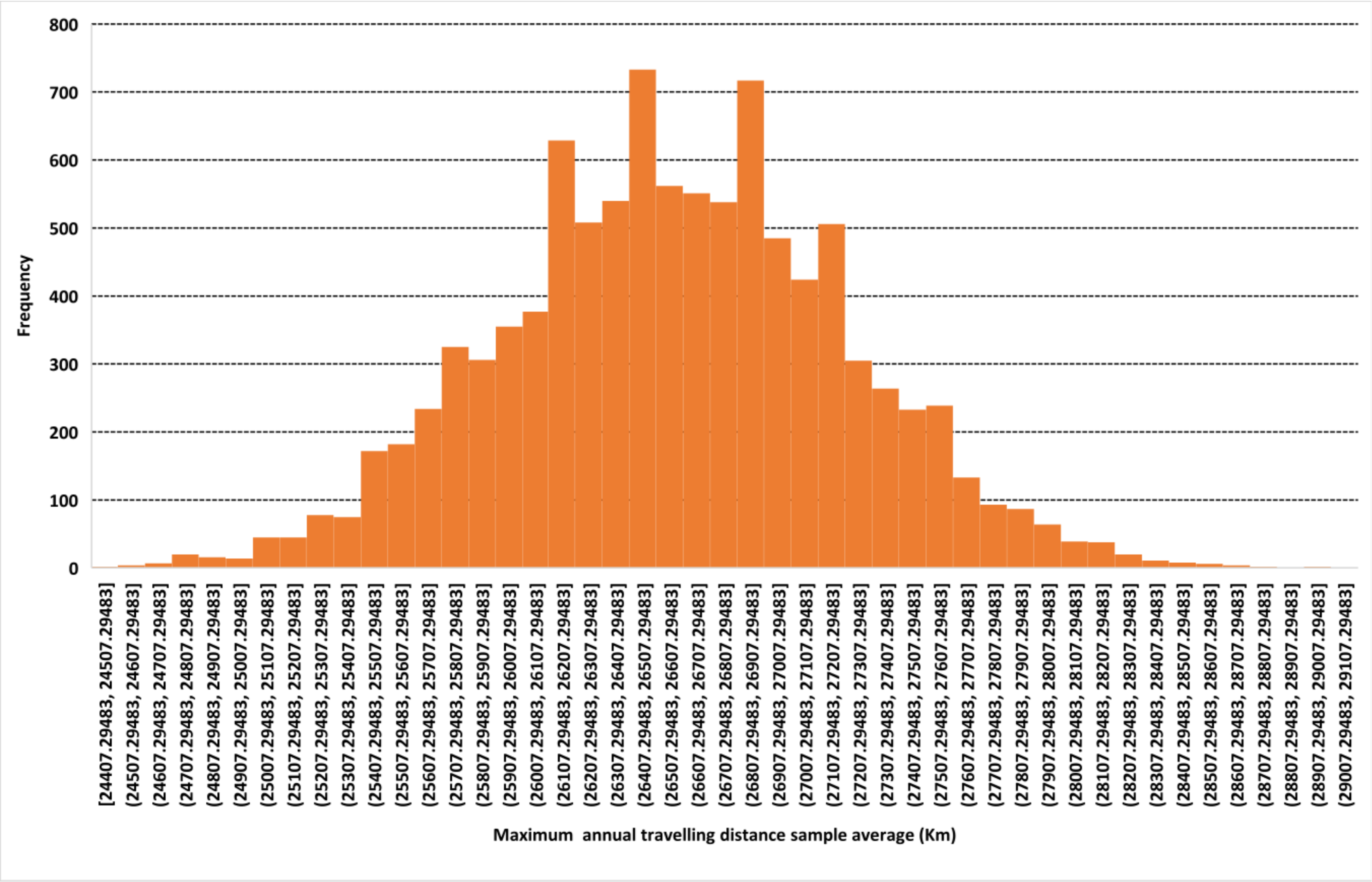

Supplementary Fig. 7 | Maximum annual traveling distance sample average histogram. n=10,000 samples

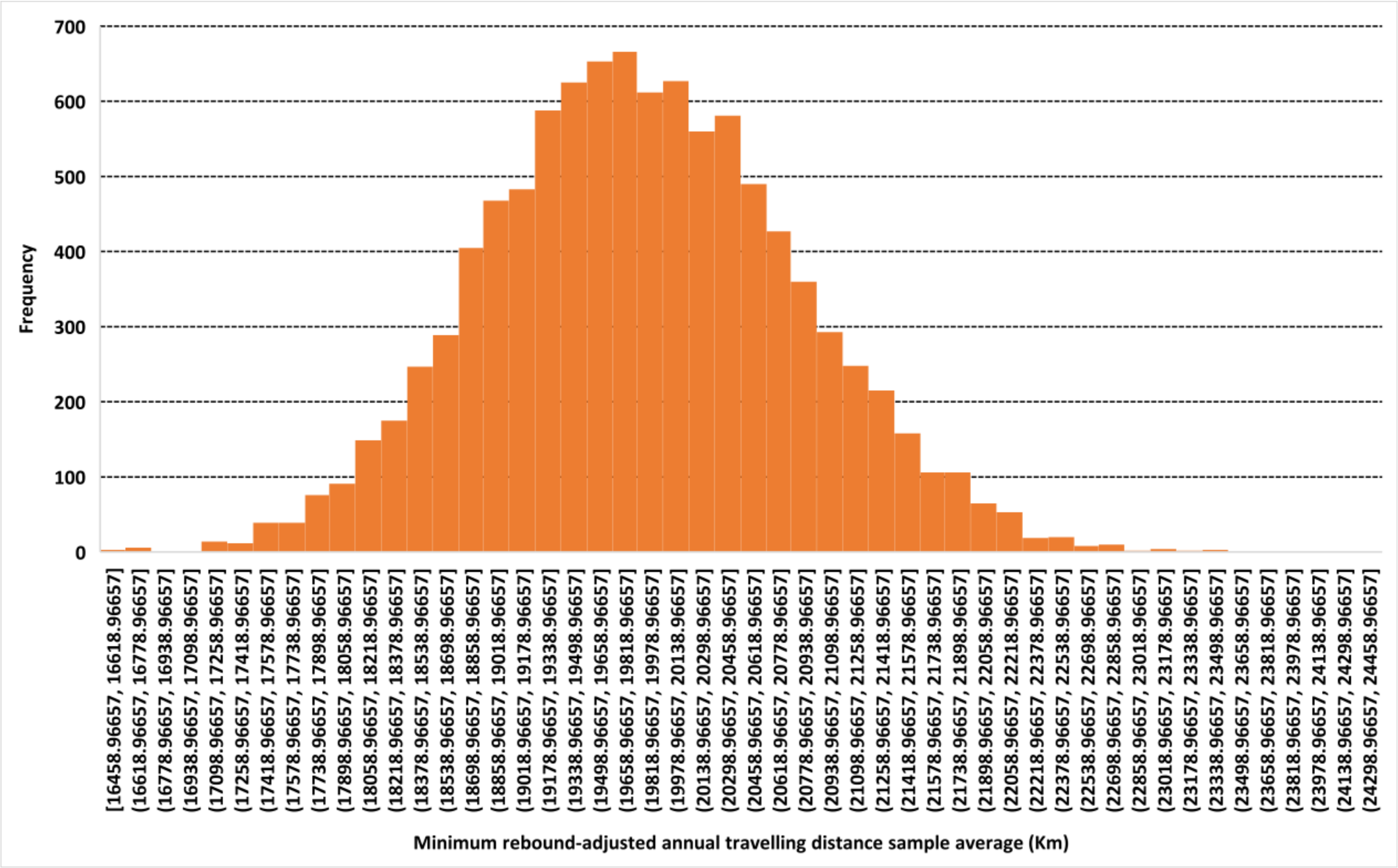

Supplementary Fig. 8 | Minimum rebound-adjusted annual traveling distance sample average histogram. n=10,000 samples

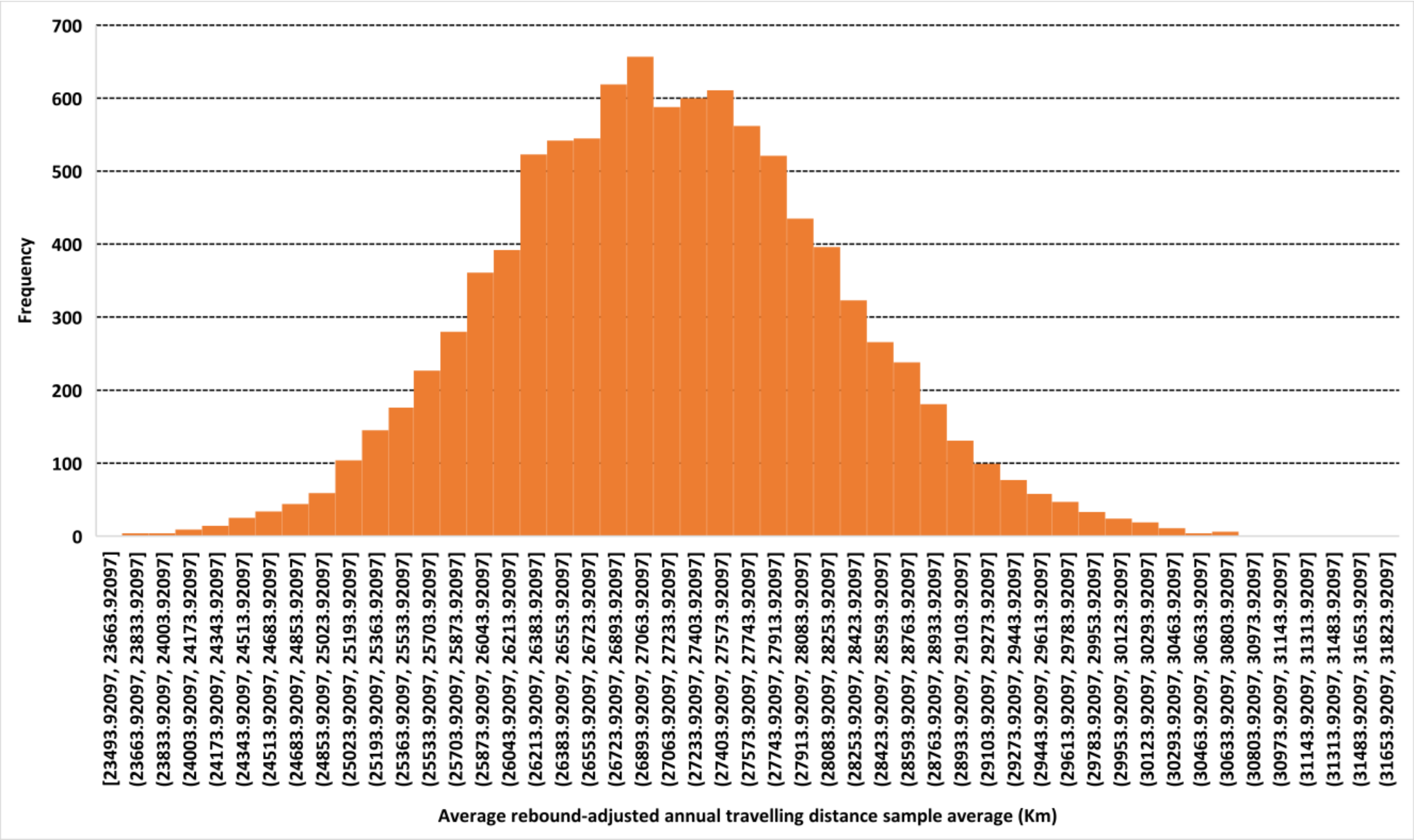

Supplementary Fig. 9 | Average rebound-adjusted annual traveling distance sample average histogram. n=10,000 samples

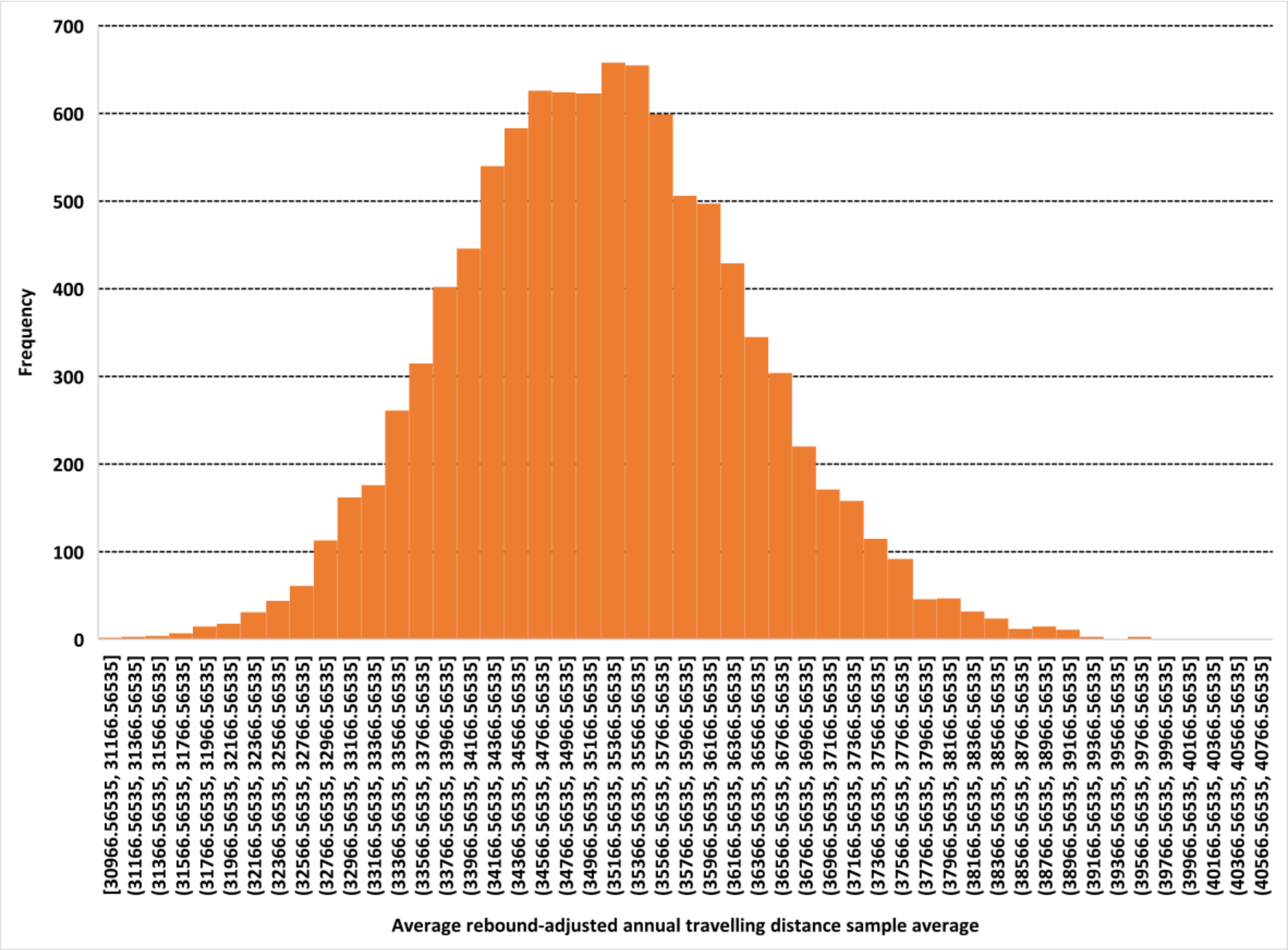

Supplementary Fig. 10 | Maximum rebound-adjusted annual traveling distance sample average histogram. n=10,000 samples

2.2 Generalized Linear Models

The generalized linear model adds a link function to the general linear model that connects the dependent variable to the components and covariates. Three generalized linear models with a 95% confidence interval are employed to investigate the significant categories affecting the dependent variables. The dependent variables for the three models are the rebound effect, annual traveling distance, and autonomous vehicle background knowledge respectively. The three models share the same independent variables including age, marital status, employment, level of education, income, number of adults per household, number of cars per household, and driving experience. Supplementary Supplementary Table ***6*** summarizes our three models' results. Firstly, households 3 to 4 with individuals who are 18 years or older were found to have a significant effect on the rebound effect. In other terms, more crowded households are likely to travel more when they adopt autonomous vehicles when compared to households with fewer adults. This was demonstrated clearly when the average rebound-adjusted annual traveling distances set was considered, households with 3 to 4 individuals had the highest average rebound effect value of 1.234 while 1-2 and 5 and over had average rebound effect values of 1.22 and 1.233 respectively. This value of 1.234 indicates that households with 3 to 4 individuals are anticipated to travel additional distances due to autonomy with an average value of 23.4% increase relevant to their annual traveling distances. It is noted that this is almost identical to the increase anticipated to be undergone by crowder households of 5 and over adults.

As for the annual traveling distance, both full-time and part-time employees are likely to travel more when compared to other categories such as students and others which includes housewives and unemployed people. This can be explained by stating that both categories represent people who must attend their jobs. One other possible explanation would be the climate of Qatar. In a study on this matter in the period between March 1999 and January 2014 conducted by [39], the daytime means maximum air temperature was over 50 °C in most of the two months with a minimum temperature of over 30°C. The situation of climate in Qatar is exacerbated by the global warming & climate change. This leads people to prefer to use their cars when compared to public transportation which sometimes requires people to walk to reach the public transportation stations. Hot climate conditions increase travel demand, thus people with responsibilities/duties are found to be the first segment of potential early adopters of autonomous vehicles. regarding the bachelor's degree. Regarding the two driving experience categories of 0. Firstly, the people with no driving experience would represent the category of people who don't drive at all or have their drivers readily available to transport them. This provided convenience of transportation could lead to more traveling. Finally, the autonomous vehicles background knowledge model concluded with no significant categories among the respondents who are more likely to have better background knowledge.

Supplementary Table 6 | Generalized linear models results. 95% confidence interval. A category is considered significant if its significance level is < 0.05.

Model	Significant Categories	Significance level	The goodness of fit test variable
Rebound Effect	Individuals – 3 to 4	0.013	0.72
Annual Traveling Distance	Employment – Full-time employee	0.027	0.785
	Employment – Part-time employee	0.031	
	Education – Bachelor's degree	0.025	
	Experience – 0 years	0.02	
Autonomous Vehicle Background Knowledge	-	-	0.789

2.3 Autonomous Vehicles Adoption Perceptions

This stage defines a crucial step in autonomous vehicle adoption due to its importance in identifying the public's opinions about autonomous vehicles and what relevant possible concerns are to be addressed properly. Four proposed benefits of autonomous vehicles were investigated which include reducing congestion on the roadways, reducing fuel consumption, reducing travel time, and reducing parking costs as shown in Supplementary Table 7**Error! Reference source not found.**. The most agreed benefit among respondents was reducing fuel consumption which had 63.6% positive opinions about it, 22.7% neutral, and 13.7% negative opinions. On the other hand, reducing traveling time was the least accepted autonomous vehicle benefit with only 47% positive opinions. Secondly, the proposed concerns include securing the autonomous driving system from computer hackers, the possibility of accidents between regular cars and autonomous vehicles, an increase in the maintenance cost of updating the computer system of the autonomous vehicle, and autonomous vehicle's performance in harsh environments introduced in Supplementary Table 8. The respondent's greatest concern was the possibility of accidents between autonomous vehicles and regular cars with a 78.1% agreement. However, autonomous vehicles' ability to work in a harsh environment was the least concern of the respondents with 64.9% doubting the harsh environment effect on autonomous vehicles while 12.4% were skeptical of the environment's effect on autonomous vehicles' performance.

Finally, after being informed of the advantages and disadvantages of autonomous vehicles, 76.4% of the respondents declared they are willing to shift to using them when they become available. On the other hand, 23.6% opposed their use in Qatar. To investigate which categories of people are more likely to adopt autonomous vehicles, it was concluded that old adults (46 years old and over) had

the highest rate of people willing to adopt autonomous vehicles with 85.9% positive implications about autonomous vehicles when compared to all other age groups. When related to their annual traveling distance, people traveling less than 20,000 kilometers were the category that had the highest percentage of switching to autonomous vehicles with 79.5%. As for income, people with monthly incomes of over 40,000 QAR, were the most open to switching to autonomous vehicles with 83.7% agreement among them. When we seek the number of cars per household as an indication for switching to autonomous vehicles, households with 3 to 4 cars are the category with the most positive adoption perspectives with 82%. Also, one important factor to consider in autonomous vehicle adoption is the people's background knowledge of autonomous vehicles which played a major impact in affecting their openness to switching with 94.4% of people with strong backgrounds willing to adopt autonomous vehicles. One important factor for the adoption of autonomous vehicles would be the price for the consumer. Using combinations of bodies, Li-Ion batteries, and autonomous system prices, it is anticipated that the average price of a fully autonomous vehicle will be around $51600.

Supplementary Table 7 | Autonomous vehicles proposed benefits summary. n= 330 samples

	Positive (%)	Neutral (%)	Negative (%)
Reducing congestion on the roadways	47.6	31.8	20.6
Reducing fuel consumption	63.7	22.7	13.6
Reducing travel time	47	33	20
Reducing parking cost	47.9	32.4	19.7

Supplementary Table 8 | Autonomous vehicles adoption concerns summary. n= 330 samples

	Positive (%)	Neutral (%)	Negative (%)
Securing autonomous driving systems from computer hackers	69.1	23	7.9
Possibility of accidents between regular cars and AVs	78.1	17	4.9
Increase in maintenance cost of updating computer system of AV	69.1	21.5	9.4
AV performance in harsh environments	64.9	22.7	12.4

3. Life Cycle Assessment Supporting Information

LCA is a method for examining the environmental implications of a product over its life cycle. LCA can reveal opportunities in the value chain of a product or a service and can provide potential environmental reduction strategies, enhance strategic planning, and educate public policy. LCAs allow practitioners to compare diverse items, allowing them to make better-informed judgments [10]. LCAs

may also be used to improve a product, process, or system design. For instance, an LCA might identify parts of the manufacturing process that have a significant environmental effect, allowing for the construction and comparison of alternative production pathways.

3.1 Manufacturing Phase

In this phase, the Manufacturing phase is assumed to comprise three processes: Manufacturing, Shipping, and Rebound effect. Nine countries including China, Japan, India, South Korea, the United States, Mexico, Germany, Turkey, and France are analyzed. These countries are represented by twelve different sedan car brands originating in those countries. Japan, the United States, and Germany have two brands each while the remaining countries are represented with only one brand each. To start, the manufacturing process comprises of manufacturing three main components of autonomous vehicles as follows:

1- Vehicle body
2- Lithium-Ion battery
3- Autonomous system components

For our case, Stochastic decision analysis is necessary due to the inherent geographical uncertainty in the production and assembly of components for autonomous vehicles. This is because we are dealing with untested, futuristic technology. Our research considers all possible manufacturing combinations for autonomous cars and analyzes nine countries where the vehicle's body, li-ion battery, and components of the autonomous system might be built. The environmental effects of constructing a vehicle's body in one country might vary greatly from those in another due to variations in technology, availability of raw materials, and energy sources. For this reason, the research presented here is essential to the future of the autonomous electric car. This exhaustive analysis takes into consideration these variances and evaluates how they impact the development of autonomous battery electric vehicles. The results of this research may serve to influence crucial choices affecting the manufacture of vehicles by putting light on the possible effects of different production configurations. This study was conducted to throw light on these potential outcomes. The results shown in the main body of the research paper show the average estimation based on those twelve sedan brands considered. By taking the average of the data, any outliers or extreme results in a single country can be less of a factor. This gives a more consistent and accurate picture of the industrial environment. Using the average of the nine countries also helps to account for the variety and unpredictability of the data, since each country has its own unique technological, raw material, and energy source problems and opportunities. By combining the results from several countries, the research can get a more complete picture of the environment for making autonomous electric vehicles and the problems and opportunities that come with it.

Starting with the vehicle body, Autonomous vehicles are assumed to have identical vehicle bodies when compared to non-autonomous battery electric vehicles. In other words, the emissions associated with both cases will be similar in this matter. Secondly, Lithium-Ion Battery (LIB) currently has many chemistries associated with the materials used in the battery. The most common chemistries in the industry include Lithium Cobalt Oxide (LCO), Lithium Iron Phosphate (LFP), and Nickel Manganese Cobalt (NMC). Those three chemistries accounted for over %83 of the market share of LIB in 2016[11]. However, LCO is not used in automobiles for some safety risks[12]. LFPs have an energy density requirement that is projected to be lower than the requirements for energy density in 2025[13]. Thus, autonomous vehicles in this study are assumed to be operated by Lithium-Ion batteries with NMC 622.

As for non-autonomous battery electric vehicles, the only difference between them in the manufacturing process is the autonomous system components which are absent from battery electric vehicles. Regarding the shipping process, autonomous vehicles are assumed to be shipped from their country of origin’s busiest port based on the annual number of containers handled[14]. The destination port is assumed to be Doha port. A fixed shipping rate of 0.4 $/ mile is used for the total shipping cost[15].

Finally, the rebound effect is considered in the manufacturing phase since it will cause increased demand for manufacturing vehicles. Since people will travel more with autonomous vehicles because of the rebound effect, their cars will deteriorate faster, and they will need to buy a new car sooner than if it has been a non-autonomous car. Having to buy cars sooner will cause the manufacturers to produce more cars resulting in more emissions when compared to non-autonomous vehicles.

Supplementary Table 9 | Autonomous vehicle components manufacturing checklist. This table summarizes which countries produce which parts within autonomous vehicles bodies, li-ion batteries, and autonomous system components.

Component / Country	Vehicle body	Lithium-Ion Battery	LIDAR	Ultrasonic sensors	InfraRed sensors	Cameras	CAN bus	ACC
China	✓	✓	✓	✓	✓	✓	✓	✓
Japan	✓	✓	✓	✓	✓	✓		✓
India	✓	✓		✓	✓			✓
South Korea	✓	✓	✓	✓	✓	✓	✓	✓
United States	✓	✓	✓	✓	✓	✓	✓	✓
Mexico	✓	✓			✓			
Germany	✓	✓	✓	✓	✓	✓	✓	✓
Turkey	✓	✓		✓				✓
France	✓	✓	✓	✓	✓	✓	✓	✓

[16–48]

Supplementary Table 10 | Autonomous vehicle components specifications & costs [1]. This table summarizes the costs of autonomous vehicles bodies, li-ion batteries, and autonomous system components for the twelve car brands considered.

Country	Brand reference name	Fuel efficiency (kWh/km)	Car MSRP ($, including battery)	Car MSRP ($, excluding battery)	Car Production Cost ($, excluding battery) *	Battery capacity (kWh)	Battery MSRP ($)	Battery Production Cost ($)	LIDAR MSRP ($)	Ultrasonic Sensors MSRP ($)	Infrared Sensors MSRP ($)	Cameras MSRP ($)	RADAR MSRP ($)	Processor & Software MSRP ($)	ACC MSRP ($)	Total Autonomous System MSRP ($)
China	China	0.14	37848	30839	24671	53.1	7009	4906	1898	112	1853	1052	500	1522	1789	8726
Japan	Japan 1	0.19	36278	26853	21482	71.4	9425	6598	4122	150	2214	1205	1000	1745	2112	12548
Japan	Japan 2	0.174	28361	20177	16142	62	8184	5729	2738	146	2214	1205	2276	1745	1800	12124
India	India	0.0968	22088	18102	14482	30.2	3986	2790	600	105	1748	1007	1848	1458	1696	8462
South Korea	Korea	0.186	40418	32762	26210	58	7656	5359	1740	130	2124	1167	3500	1690	2032	12383
United States	US 1	0.2	42895	29867	23894	98.7	13028	9120	1000	-	-	1330	2833	1925	3200	10288
United States	US 2	0.17	49440	38616	30893	82	10824	7577	3470	130	2507	1330	2833	1925	2374	14569
Mexico	Mexico	0.1125	28678	26302	21042	18	2376	1663	1300	111	1828	1041	1954	1507	1767	9508
Germany	Germany 1	0.182	42472	35212	28170	55	7260	5082	8270	83	2300	1242	3263	1798	2189	19145
Germany	Germany 2	0.172	64611	53536	42829	83.9	11075	7753	4618	141	2300	1242	3040	1798	1700	14839
Turkey	Turkey	0.18	44402	32522	26018	90	11880	8316	3790	111	1835	1044	1964	1511	1773	12028
France	France	0.165	35034	27814	22251	54.7	7220	5054	1000	185	2203	1201	3380	1738	2102	11809

-Body: 20% profit margin [49], Battery: 30% profit margin (assumed), Autonomous system components 30% profit margin (assumed)

Supplementary Table 11 | Autonomous vehicle components specifications & costs reference[2]. This table shows the references for the costs of autonomous vehicles bodies, li-ion batteries, and autonomous system components for the twelve car brands considered.

Country	Brand reference name	Fuel efficiency	Car MSRP ($, including battery)	Car weight	Battery capacity (kWh)	Battery MSRP ($)	LIDAR	Ultrasonic Sensors	Infrared Sensors	Cameras	RADAR	Processor & Software	ACC
China	China	[50]	[50]	[50]	[50]	[51]	[52]	*1	*2	*3	[53]	*5	*6
Japan	Japan 1	[54]	[55]	[54]	[54]	[51]	[56] 150 m	[57]	*2	*3	[58]	*5	*6
Japan	Japan 2	[54]	[59]	[60]	[54]	[51]	[61]	[62]	*2	*3	[63]	*5	[64]
India	India	[65]	[65]	[65]	[65]	[51]	[66]	*1	*2	*3	*4	*5	*6
South Korea	Korea	[54]	[67]	[54]	[54]	[51]	[68]	*1	*2	*3	[69]	[70–73]	*6
United States	US 1	[54]	[74]	[75]	[54]	[51]	[76]	- Included in ACC [77]	*2	[78–80] assumed 4 cameras	*4	*5	[81]
United States	US 2	[54]	[82]	[54]	[54]	[51]	[83]	[84]	*2	*3	*4	*5	*6
Mexico	Mexico	[85]	[86]	[87]	[87]	[51]	[88]	*1	*2	*3	*4	*5	*6
Germany	Germany 1	[54]	[89]	[54]	[54]	[51]	[90]	[91]	*2	*3	[92]	*5	*6
Germany	Germany 2	[54]	[93]	[54]	[54]	[51]	*	*1	[94]	*3	[95]	*5	[96]

Turkey	Turkey	[97]	[98]	Estimated	[97]	[51]	[99]	*1	*2	*3	*4	*5	*6
France	France	[54]	[100]	[54]	[54]	[51]	[101]	[102]	*2	*3	[103]	*5	*6

* Estimate component price in country Y based on other countries' component prices (Country X) using $Price\ in\ country\ Y = (0.75 \times Price\ in\ country\ X) + (0.25\ \times \left(\frac{GDP\ per\ capita\ for\ country\ Y}{GDP\ per\ capita\ for\ country\ X}\right) \times Price\ in\ country\ X)$ (5). Estimating a component's price in country Y using multiple prices from country X is as follows:

A- Estimate component price for country Y using country X information.

B- Repeat (A) for all countries' component prices X

C- Round prices to a whole number obtained in (B), then their average is the components price for country Y using multiple components prices from countries X.

$$Price\ in\ country\ Y = (0.75 \times Price\ in\ country\ X) + (0.25\ \times \left(\frac{GDP\ per\ capita\ for\ country\ Y}{GDP\ per\ capita\ for\ country\ X}\right) \times Price\ in\ country\ X) \quad (5)$$

* LIDAR price estimation for GERMANY 2 using the given prices from CHINA, Japan 1, Japan 2, INDIA, Korea, US 1, US 2, Mexico, Germany 1, TURKEY, and France:

= Average (0.75 × 1898 + 0.25 × (64530 ÷ 10550) × 1898), (0.75 × 4122 + 0.25 × (64530 ÷ 40360) × 4122), (0.75 × 2338 + 0.25 × (64530 ÷ 40360) × 2338), (0.75 × 600 + 0.25 × (64530 ÷ 1920) × 600), (0.75 × 1740 + 0.25 × (64530 ÷ 32960)× 1740), (0.75 × 1000 + 0.25 × (64530 ÷ 64530) × 1000), (0.75 × 3470 + 0.25 × (64530 ÷ 8480) × 3470), (0.75 × 1300 + 0.25 × (64530 ÷ 47470) × 1300), (0.75 × 8270 + 0.25 × (64530 ÷ 47470) × 8270), (0.75 × 3790 + 0.25 × (64530 ÷ 9050) × 3790), (0.75 × 1000 + 0.25 × (64530 ÷ 39480) × 1000) = 4618

*1 Ultrasonic sensor price estimation using the given prices from Japan 1, Japan 2, US 2, Germany 1, and France:

A- CHINA = Average (0.75 × 150 + 0.25 × (10550 ÷ 40360) × 150), (0.75 × 146+ 0.25 × (10550 ÷ 40360) × 146), (0.75 × 130 + 0.25 × (10550 ÷ 64530) × 130), (0.75 × 83 + 0.25 × (10550 ÷ 47470) × 83), (0.75 × 185 + 0.25 × (10550 ÷ 39480) × 185) = 112

B- INDIA = Average (0.75 × 150 + 0.25 × (1920 ÷ 40360) × 150), (0.75 × 146+ 0.25 × (1920 ÷ 40360) × 146), (0.75 × 130 + 0.25 × (1920 ÷ 64530) × 130), (0.75 × 83 + 0.25 × (1920 ÷ 47470) × 83), (0.75 × 185 + 0.25 × (1920 ÷ 39480) × 185) = 105

C- Korea = Average (0.75 × 150 + 0.25 × (32960 ÷ 40360) × 150), (0.75 × 146+ 0.25 × (32960 ÷ 40360) × 146), (0.75 × 130 + 0.25 × (32960 ÷ 64530) × 130), (0.75 × 83 + 0.25 × (32960 ÷ 47470) × 83), (0.75 × 185 + 0.25 × (32960 ÷ 39480) × 185)= 130

D- Mexico = Average (0.75 × 150 + 0.25 × (8480 ÷ 40360) × 150), (0.75 × 146+ 0.25 × (8480 ÷ 40360) × 146), (0.75 × 130 + 0.25 × (8480 ÷ 64530) × 130), (0.75 × 83 + 0.25 × (8480 ÷ 47470) × 83), (0.75 × 185 + 0.25 × (8480 ÷ 39480) × 185) = 111

E- GERMANY 2 = Average (0.75 × 150 + 0.25 × (47470 ÷ 40360) × 150), (0.75 × 146+ 0.25 × (47470 ÷ 40360) × 146), (0.75 × 130 + 0.25 × (47470 ÷ 64530) × 130), (0.75 × 83 + 0.25 × (47470 ÷ 47470) × 83), (0.75 × 185 + 0.25 × (47470 ÷ 39480) × 185) = 141

F- TURKEY = Average (0.75 × 150 + 0.25 × (9050 ÷ 40360) × 150), (0.75 × 146+ 0.25 × (9050 ÷ 40360) × 146), (0.75 × 130 + 0.25 × (9050 ÷ 64530) × 130), (0.75 × 83 + 0.25 × (9050 ÷ 47470) × 83), (0.75 × 185 + 0.25 × (9050 ÷ 39480) × 185) = 111

2* Infrared sensors price estimation using the given price from GERMANY 2:

A- CHINA = 0.75 × 2300 + 0.25× (10550 ÷ 47470) × 2300 = 1853
B- Japan 1 = 0.75 × 2300 + 0.25 × (40360 ÷ 47470) × 2300= 2214
C- Japan 2 = 0.75 × 2300 + 0.25 × (40360 ÷ 47470) × 2300= 2214
D- INDIA = 0.75 × 2300 + 0.25 × (1920 ÷ 47470) × 2300= 1748
E- Korea = 0.75 × 2300 + 0.25 × (32960 ÷ 47470) × 2300 = 2124
F- US 1 = 0.75 × 2300 + 0.25 × (64530 ÷ 47470) × 2300 = 2507
G- US 2 = 0.75 × 2300 + 0.25 × (64530 ÷ 47470) × 2300 = 2507
H- Mexico = 0.75 × 2300 + 0.25 × (8480 ÷ 47470) × 2300= 1828
I- Germany 1 = 0.75 × 2300 + 0.25 × (47470 ÷ 47470) = 2300
J- TURKEY = 0.75 × 2300 + 0.25 × (9050 ÷ 47470) × 2300= 1835
K- France = 0.75 × 2300 + 0.25 × (39480 ÷ 47470) × 2300= 2203

3* Camera price estimation using the given price from US 1:

A- CHINA = 0.75 × 1330 + 0.25× (10550 ÷ 64530) × 1330 = 1052
B- Japan 1 = 0.75 × 1330 + 0.25 × (40360 ÷ 64530) × 1330= 1205
C- Japan 2 = 0.75 × 1330 + 0.25 × (40360 ÷ 64530) × 1330= 1205
D- INDIA = 0.75 × 1330 + 0.25 × (1920 ÷ 64530) × 1330= 1007
E- Korea = 0.75 × 1330 + 0.25 × (32960 ÷ 64530) × 1330 = 1167
F- US 2 = 0.75 × 1330 + 0.25 × (64530 ÷ 64530) × 1330 = 1330
G- Mexico = 0.75 × 1330 + 0.25 × (8480 ÷ 64530) × 1330= 1041
H- Germany 1 = 0.75 × 1330 + 0.25 × (47470 ÷ 64530) × 1330= 1242
I- GERMANY 2 = 0.75 × 1330 + 0.25 × (47470 ÷ 64530) × 1330= 1242
J- TURKEY = 0.75 × 1330 + 0.25 × (9050 ÷ 64530) × 1330= 1044
K- France = 0.75 × 1330 + 0.25 × (39480 ÷ 64530) × 1330= 1201

4* Radar price estimation using the given prices from CHINA, Japan 1, Japan 2, Korea, Germany 1, GERMANY 2, and France:

A- INDIA = Average (0.75 × 500 + 0.25 × (1920 ÷ 10550) × 500), (0.75 × 1000 + 0.25 × (1920 ÷ 40360) × 1000), (0.75 × 2276 + 0.25 × (1920 ÷ 40360) × 2276), (0.75 × 3500 + 0.25 × (1920 ÷ 32960) × 3500), (0.75 × 3263 + 0.25 × (1920 ÷ 47470) × 3263), (0.75 × 3040 + 0.25 × (1920 ÷ 47470) × 3040), (0.75 × 3380 + 0.25 × (1920 ÷ 39480) × 3380) = 1848
B- US 1 = Average (0.75 × 500 + 0.25 × (64530 ÷ 10550) × 500), (0.75 × 1000 + 0.25 × (64530 ÷ 40360) × 1000), (0.75 × 2276 + 0.25 × (64530 ÷ 40360) × 2276), (0.75 × 3500 + 0.25 × (64530 ÷ 32960) × 3500), (0.75 × 3263 + 0.25 × (64530 ÷ 47470) × 3263), (0.75 × 3040 + 0.25 × (64530 ÷ 47470) × 3040), (0.75 × 3380 + 0.25 × (64530 ÷ 39480) × 3380) = 2833
C- US 2 = Average (0.75 × 500 + 0.25 × (64530 ÷ 10550) × 500), (0.75 × 1000 + 0.25 × (64530 ÷ 40360) × 1000), (0.75 × 2276 + 0.25 × (64530 ÷ 40360) × 2276), (0.75 × 3500 + 0.25 × (64530 ÷ 32960) × 3500), (0.75 × 3263 + 0.25 × (64530 ÷ 47470) × 3263), (0.75 × 3040 + 0.25 × (64530 ÷ 47470) × 3040), (0.75 × 3380 + 0.25 × (64530 ÷ 39480) × 3380) = 2833
D- Mexico = Average (0.75 × 500 + 0.25 × (8480 ÷ 10550) × 500), (0.75 × 1000 + 0.25 × (8480 ÷ 40360) × 1000), (0.75 × 2276 + 0.25 × (8480 ÷ 40360) × 2276), (0.75 × 3500 + 0.25 × (8480 ÷ 32960) × 3500), (0.75 × 3263 + 0.25 × (8480 ÷ 47470) × 3263), (0.75 × 3040 + 0.25 × (8480 ÷ 47470) × 3040), (0.75 × 3380 + 0.25 × (8480 ÷ 39480) × 3380) = 1954
E- TURKEY = Average (0.75 × 500 + 0.25 × (9050 ÷ 10550) × 500), (0.75 × 1000 + 0.25 × (9050 ÷ 40360) × 1000), (0.75 × 2276 + 0.25 × (9050 ÷ 40360) × 2276), (0.75 × 3500 + 0.25 × (9050 ÷ 32960) × 3500), (0.75 × 3263 + 0.25 × (9050 ÷ 47470) × 3263), (0.75 × 3040 + 0.25 × (9050 ÷ 47470) × 3040), (0.75 × 3380 + 0.25 × (9050 ÷ 39480) × 3380) = 1964

5* Processor & Software:

US 2: (Processor $1,500, DSRC $73, V2X $222, and GPS $130). Total: $1925

price estimation using the given price from US 2:

A- CHINA= 0.75 × 1925 + 0.25× (10550 ÷ 64530) × 1925 = 1522
B- Japan 1= 0.75 × 1925 + 0.25 × (40360 ÷ 64530) × 1925 = 1745
C- Japan 2= 0.75 × 1925 + 0.25 × (40360 ÷ 64530) × 1925 = 1745
D- INDIA= 0.75 × 1925 + 0.25 × (1920 ÷ 64530) × 1925 = 1458
E- Korea= 0.75 × 1925 + 0.25 × (32960 ÷ 64530) × 1925 = 1690
F- US 1= 0.75 × 1925 + 0.25 × (64530 ÷ 64530) × 1925 = 1925
G- Mexico= 0.75 × 1925 + 0.25 × (8480 ÷ 64530) × 1925 = 1507
H- Germany 1= 0.75 × 1925 + 0.25 × (47470 ÷ 64530) × 1925 = 1798
I- GERMANY 2= 0.75 × 1925 + 0.25 × (47470 ÷ 64530) × 1925 = 1798
J- TURKEY= 0.75 × 1925 + 0.25 × (9050 ÷ 64530) × 1925 = 1511
K- France= 0.75 × 1925 + 0.25 × (39480 ÷ 64530) × 1925 = 1738

6* Adaptive cruise control cost estimation using given prices from Japan 2, US 1, and GERMANY 2:

A- CHINA = Average (0.75 × 1800 + 0.25 × (10550 ÷ 40360) × 1800), (0.75 × 3200 + 0.25 × (10550 ÷ 64530) × 3200), (0.75 × 1700 + 0.25 × (10550 ÷ 47470) × 1700) = 1789
B- Japan 1 = Average (0.75 × 1800 + 0.25 × (40360 ÷ 40360) × 1800), (0.75 × 3200 + 0.25 × (40360 ÷ 64530) × 3200), (0.75 × 1700 + 0.25 × (40360 ÷ 47470) × 1700) = 2112
C- INDIA = Average (0.75 × 1800 + 0.25 × (1920 ÷ 40360) × 1800), (0.75 × 3200 + 0.25 × (1920 ÷ 64530) × 3200), (0.75 × 1700 + 0.25 × (1920 ÷ 47470) × 1700) = 1696
D- Korea = Average (0.75 × 1800 + 0.25 × (32960 ÷ 40360) × 1800), (0.75 × 3200 + 0.25 × (32960 ÷ 64530) × 3200), (0.75 × 1700 + 0.25 × (32960 ÷ 47470) × 1700) = 2032
E- US 2 = Average (0.75 × 1800 + 0.25 × (64530 ÷ 40360) × 1800), (0.75 × 3200 + 0.25 × (64530 ÷ 64530) × 3200), (0.75 × 1700 + 0.25 × (64530 ÷ 47470) × 1700) = 2374
F- Mexico = Average (0.75 × 1800 + 0.25 × (8480 ÷ 40360) × 1800), (0.75 × 3200 + 0.25 × (8480 ÷ 64530) × 3200), (0.75 × 1700 + 0.25 × (8480 ÷ 47470) × 1700) = 1767
G- Germany 1 = Average (0.75 × 1800 + 0.25 × (47470 ÷ 40360) × 1800), (0.75 × 3200 + 0.25 × (47470 ÷ 64530) × 3200), (0.75 × 1700 + 0.25 × (47470 ÷ 47470) × 1700) = 2189

H- TURKEY = Average (0.75 × 1800 + 0.25 × (9050 ÷ 40360) × 1800), (0.75 × 3200 + 0.25 × (9050 ÷ 64530) × 3200), (0.75 × 1700 + 0.25 × (9050 ÷ 47470) × 1700) = 1773

I- France = Average (0.75 × 1800 + 0.25 × (39480 ÷ 40360) × 1800), (0.75 × 3200 + 0.25 × (39480 ÷ 64530) × 3200), (0.75 × 1700 + 0.25 × (39480 ÷ 47470) × 1700) = 2102

3.2 Operation Phase

In this phase, the emissions produced during service life are estimated. Mainly, emissions in the vehicle's operation phase come from two different parts: Well-to-Tank (WTT) and Tank-to-Wheel (TTW). Starting with WTT, it is concerned with the emerging emissions due to the production, processing, and delivery of energy. As for TTW, it represents tailpipe emissions emerging due to the vehicle operation. Autonomous vehicles aren't associated with any TTW emissions (since they are assumed to be operated by batteries and have no tailpipe emissions). For this reason, the only emissions assessed in the operation phase are for the electricity generation to charge batteries. $EGE = EGP \times GHG\ Factor \times ETL\ Factor$ () is used to calculate emissions associated with the generation of 1 kWh of electricity in Qatar.

$$EGE = EGP \times GHG\ Factor \times ETL\ Factor \quad (6)$$

Where EGE: electricity generation emission (Kg GHG); EGP: electricity generation price (constant,0.15 QAR/kWh[104]); GHG factor (kg GHG/kWh): obtained from the EXIOBASE database using electricity generation from gas as a sector for scenarios 1 and 2, electricity generation using Photovoltaics for scenarios 3 and 4, the year 2021 as a basis, and World Middle East (WM) as a reference country; ETL factor: The electricity transmission losses factor is used to compensate for 10% of energy losses from the transmission of electricity.

However, this EGE value of emissions accounts for 1 kWh only. To find emissions associated with driving 1-kilometer using autonomous vehicles, we must first quantify one of the proposed benefits of adopting autonomous vehicles which is the increased fuel efficiency due to autonomy. [105] concluded that autonomous vehicles introduce improvement in fuel efficiency when compared to non-autonomous vehicles, the improved fuel efficiency is referred to as fuel economy for this paper. The improvement is quantified to be approximately 23.6%. In this study, this value is used to compensate for this improvement as in $FEC = BEV - FE$[illegible]$(1 + FEI)$ (7). The fuel efficiency values for the twelve brands considered are taken from their perspective manufacturers. Refer to Supplementary Table ***10*** for the fuel efficiency values and

Supplementary **Table 11** for references to those values. Considering different values of fuel efficiencies for the considered sedan vehicles assists in facing the uncertainty associated with operating parameters (fuel efficiency) of an emerging technology such as

autonomous vehicles. The improvement is mirrored in all twelve brands considered, where each of the vehicles considered gains an improvement in fuel efficiency when they are assumed to be AVs.

$$FEC = \frac{BEV-FE}{(1+FEI)} \quad (7)$$

Where FEC: fuel economy (kWh/km); BEV-FE: battery electric vehicle fuel efficiency (kWh/Km); FEI: % improvement in fuel efficiency. Then, FEC is used to calculate autonomous vehicle's operation phase emissions factor for each GHG considered as in

$$AV{-}OP\ EF = EGE \times FEC$$

().

$$AV - OP\ EF = EGE \times FEC \quad (8)$$

Where AV-OP EF: autonomous vehicle operation phase emission factor.

3.3 End-of-Life Phase

The End-of-Life (EOL) phase is the subsequent phase after the vehicle has reached its maximum mileage after which it is salvaged. Generally, this phase results in a saving in emissions since recycling sends out fewer emissions when compared to mining for the materials. This is because recycling requires less energy than the mining process which indicates it requires less fossil fuel to burn for the process[106]. This outcome is resembled by negative values of emissions due to savings resulting from recycling. To start, the GREET model for the year 2021 is used to obtain what materials exist in a vehicle as well as for a Li-Ion battery. Then for the autonomous system, each part is divided into the materials it comprises. Consequently, for each material used in the autonomous vehicle, the total amount needed in a vehicle is calculated. Then, the recycling emissions saving (RES) is calculated for each material (x) as in equation (9) using GWP values for recycling (GWPR) and mining (GWPM). The recovery rate (RR) is used to compensate for the losses of the material during the recycling process.

$$\mathrm{RES}\ (x) = \frac{x\ in\ \mathrm{AV}}{\mathrm{RR}(x)} \times \mathrm{GWPR}(x) - x\ in\ \mathrm{AV} \times \mathrm{GWPM}(x) \quad (9)$$

Next is the global warming potential (GWP) for the considered greenhouse gases is calculated to come up with a unified measure of emissions rather than expressing the emissions of each greenhouse gas by itself as follows in equation (10).

$$\mathrm{LC\ GWP} = \mathrm{LC\ CO2} + \mathrm{LC\ CH4} \times \mathrm{CH4\ GWP} + \mathrm{LC\ N2O} \times \mathrm{N2O\ GWP} \quad (10)$$

Where LC GWP: life cycle global warming potential (Kg CO2-Eq.); LC CO2; life cycle carbon dioxide emissions; LC CH4: life cycle methane emissions; CH4 GWP: methane global warming potential (27.9) 107; LC N2O: life cycle nitrous oxide emissions; N2O GWP: nitrous oxide global warming potential (273) [107].

3.4 Monte Carlo Simulation

Uncertainty in life cycle assessment influences the model's reliability and is one of its main challenges[108]. The uncertainty stems from three main sources: parameter, scenario, and model[109]. To start, parameter uncertainty comes from inherent variability in the sampled population, uncertainty in seen or measured values, and data quality uncertainty. Scenario uncertainty arises from normative choices made throughout the scenario-building process, such as those regarding the functional unit and the time horizon. Lastly, model uncertainty is due to the underlying structure of the models and the mathematical linkages that describe them. Addressing the uncertainty involves determining the total uncertainty of the conclusion based on the uncertainty of all the parameters and model selections of the simulated product system, and then generating a confidence interval for the results. the simulation parameters are shown in Supplementary Table 12 while Supplementary Table 13 shows the summary of the simulation results.

Supplementary Table 12 | Monte Carlo simulation parameters for given scenarios. This table shows each input variable distribution for different life cycle variables among the four scenarios considered in this study.

		Scenarios			
Input/Output	Variable Name	Scenario 1	Scenario 2	Scenario 3	Scenario 4
Input 1	Manufacturing emissions (ton CO2-Eq)	Burr12(0,14.338,7.9189,0.76653)	Burr12(0,2.383,7.3676,0.84094)	Burr12(0,14.338,7.9189,0.76653)	Burr12(0,2.383,7.3676,0.84094)
Input 2	Rebound effect (%)	-	Normal(1.26,0.0625)	-	Normal(1.26,0.0625)
Input 3	Fuel efficiency (kWh/km)	Dagum(0,0.19557,49.105,0.098342)	Dagum (0,0.15822,49.105,0.098342)	Dagum(0,0.19557,49.105,0.098342)	Dagum (0,0.15822,49.105,0.098342)
Input 4	Operation phase constant	119.2156092	119.2156092	23.0441544	23.0441544
Input 5	End-of-Life emissions (t CO2-Eq)	Fréchet (-13.0133,5.7793,5.7732)	Fréchet (-13.8492,6.4315,6.1543)	Fréchet (-13.0133,5.7793,5.7732)	Fréchet (-13.8492,6.4315,6.1543)
Output	Life-Cycle Global Warming	(Manufacturing emissions) + (Fuel efficiency × Operation phase constant) + (End-of-Life emissions)	(Manufacturing emissions × Rebound effect) + (Fuel economy ×	(Manufacturing emissions) + (Fuel efficiency × Operation phase constant) + (End-of-Life emissions)	(Manufacturing emissions × Rebound effect) + (Fuel economy ×

Potential (tCO_2-Eq.)	Operation phase constant) + (End-of-Life emissions)	Operation phase constant) + (End-of-Life emissions)

- $Operation\ phase\ constant = (EGP \times CO2 - factor + EGP \times CH4 - factor \times CH4 - GWP + EGP \times N2O - factor \times N2O - GWP) \times (1000\ (per\ kilometer \rightarrow total) \times \frac{ETL}{1000000(gram \rightarrow ton)} * \frac{300{,}000\ kilometers}{1{,}000{,}000\ gCO2-Eq.}).$
- Where EGP: electricity generation price: 0.15 QAR/kWh; CO2-factor (Scenarios 1,2): 9582237.199 KgCO2/ M€; CH4-factor(Scenarios 1,2): 1588.725084 KgCH4/ M€; CH4-GWP: 27.9; N2O-factor (Scenarios 1,2): 25.72143439 KgN2O/ M€; CO2-factor (Scenarios 3,4): 9582237.199 KgCO2/ M€; CH4-factor(Scenarios 3,4): 1588.725084 KgCH4/ M€; CH4-GWP: 27.9; N2O-factor (Scenarios 3,4): 25.72143439 KgN2O/ M€; N2O-GWP: 273; ETL: electricity transmission losses factor: 1.1.

Supplementary Table 13 | Monte Carlo simulation results. n=10,000 samples

Scenario	Scenario 1	Scenario 2	Scenario 3	Scenario 4
Mean (t CO2-Eq)	28.669	36.773	13.028	24.119
Standard deviation (t CO2-Eq)	5.801	8.59	4.753	8.185
Interquartile range (t CO2-Eq)	6.589	9.738	5.486	9.166
Skewness	0.065348	1.42754	1.58115	1.62714
Kurtosis	3.29811	7.43156	6.69035	7.46002

Supplementary Supplementary Fig. ***11*** shows the breakdown of each phase in the life cycle for scenarios 3 and 4 using Photovoltaics.

(a) **(b)**

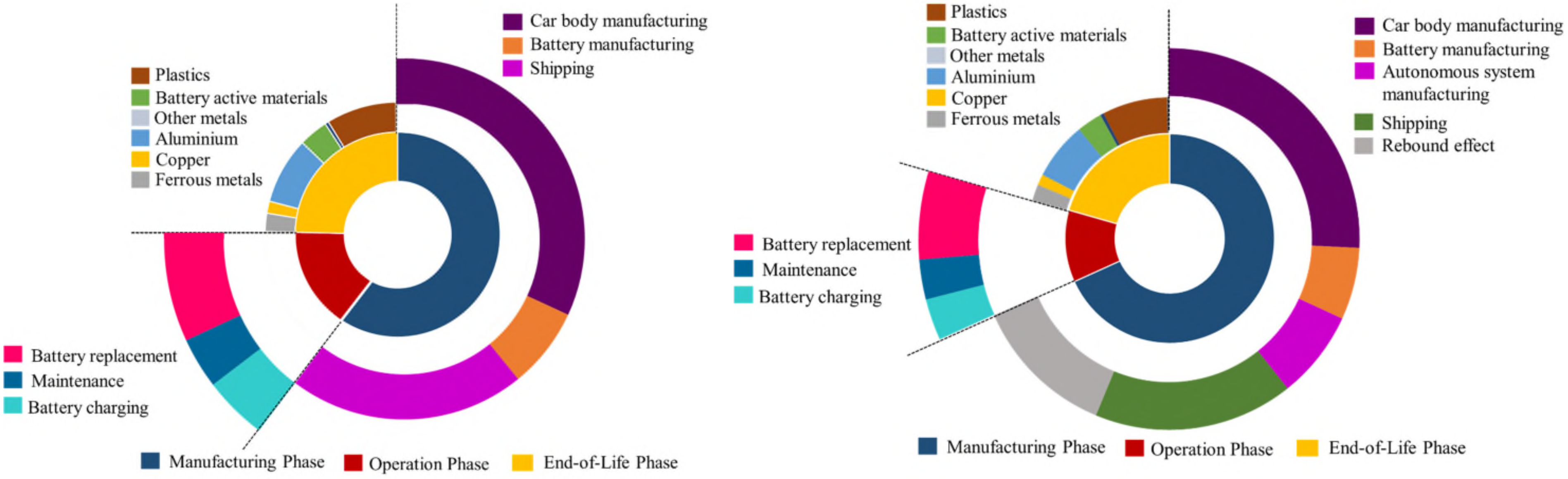

Supplementary Fig. 11 | Breakdown of life cycle emissions. **a,** Scenario 3. **b,** Scenario 4. This figure aims to provide a breakdown of life cycle emissions by phase and process simultaneously.

Supplementary Table 14 | Manufacturing, operation, and end-of-life phases cost combinations summary. n=10,000 samples.

Statistical measure	Value
Mean	51579.5
Standard error	233.5

Median	50648
Mode	49876
Standard deviation	9706.2
Sample variance	94210631.3
Kurtosis	0.842
Skewness	0.762
Range	56769
Minimum	28940
Maximum	85709
n	1728

Supplementary References

1. Automated Vehicles for Safety | NHTSA. https://www.nhtsa.gov/technology-innovation/automated-vehicles-safety.
2. Blueprint for Autonomous Urbanism: Second Edition | National Association of City Transportation Officials. https://nacto.org/publication/bau2/.
3. DSRC Technology, 802.11p standard, ITS-G5, ETSI ITS-G5, IEEE802.11p. https://auto-talks.com/technology/dsrc-technology/.
4. Connected Vehicles Technology | V2X, V2V, V2I communication | Autotalks. https://auto-talks.com/.
5. The Functional Components of Autonomous Vehicles - Expert Article | Robson Forensic. https://www.robsonforensic.com/articles/autonomous-vehicles-sensors-expert.
6. Three Sensor Types Drive Autonomous Vehicles | Fierce Electronics. https://www.fierceelectronics.com/components/three-sensor-types-drive-autonomous-vehicles.
7. Ondruš, J., Kolla, E., Vertaľ, P. & Šarić, Ž. How Do Autonomous Cars Work? *Transportation Research Procedia* **44**, 226–233 (2020).
8. Infographic: The Electrical Components of the Self-Driving Car - ASME. https://www.asme.org/topics-resources/content/infographic-the-electrical-components-of-the-self-driving-car.
9. Matzarakis, A. & Fröhlich, D. Sport events and climate for visitors—the case of FIFA World Cup in Qatar 2022. *Int J Biometeorol* **59**, 481–486 (2015).
10. Algren, M., Fisher, W. & Landis, A. E. Machine learning in life cycle assessment. *Data Science Applied to Sustainability Analysis* 167–190 (2021) doi:10.1016/B978-0-12-817976-5.00009-7.
11. Ding, Y., Cano, Z. P., Yu, A., Lu, J. & Chen, Z. Automotive Li-Ion Batteries: Current Status and Future Perspectives. *Electrochemical Energy Reviews 2019 2:1* **2**, 1–28 (2019).
12. Cho, J. Dependence of AlPO4 coating thickness on overcharge behaviour of LiCoO2 cathode material at 1 and 2 C rates. *J Power Sources* **126**, 186–189 (2004).
13. Andre, D. *et al.* Future generations of cathode materials: an automotive industry perspective. *J Mater Chem A Mater* **3**, 6709–6732 (2015).
14. List of busiest container ports - Wikipedia. https://en.wikipedia.org/wiki/List_of_busiest_container_ports.

15. Cost To Ship A Car - Easy Auto Ship | Shipping Made Easy. https://diytransport.com/cost-to-ship-a-car/.

16. Car Production by Country 2022. https://worldpopulationreview.com/country-rankings/car-production-by-country.

17. China Dominates the Lithium-ion Battery Supply Chain, but Europe is on the Rise | BloombergNEF. https://about.bnef.com/blog/china-dominates-the-lithium-ion-battery-supply-chain-but-europe-is-on-the-rise/.

18. Four Companies Leading the Rise of Lithium & Battery Technology – Global X ETFs. https://www.globalxetfs.com/four-companies-leading-the-rise-of-lithium-battery-technology/.

19. The Top 10 EV Battery Makers - IEEE Spectrum. https://spectrum.ieee.org/the-top-10-ev-battery-makers.

20. CANBusCommunications. https://www.naii.com/CANBusCommunications.

21. CAN Bus interface module - 1351 series - Curtis Instruments. https://www.directindustry.com/prod/curtis-instruments/product-199498-2336802.html.

22. CAN Bus interface module - TD5(3)31SCANFD series - MORNSUN Guangzhou Science & Technology Co.,Ltd. - industrial. https://www.directindustry.com/prod/mornsun-guangzhou-science-technology-co-ltd/product-157704-2379070.html.

23. Optionskarten | AMKmotion. https://amk-motion.com/en/node/65.

24. Autonics. https://www.autonics.com/main.

25. Automotive Imaging Sensors | Image Sensors | OMNIVISION. https://www.ovt.com/applications/automotive/.

26. Camera for the automotive industry - CV-CC214 series - Caravision Technology Inc. - for vehicles / industrial / digital. https://www.directindustry.com/prod/caravision-technology-inc/product-210337-2190317.html.

27. Top 5 Vehicle Camera Manufacturers - Verified Market Research. https://www.verifiedmarketresearch.com/blog/top-vehicle-camera-manufacturers/.

28. Top 5 Vendors in the Automotive Camera Module Market - COVID Impact | Technavio | Business Wire. https://www.businesswire.com/news/home/20200909005165/en/Top-5-Vendors-in-the-Automotive-Camera-Module-Market---COVID-Impact-Technavio.

29. Pyroelectric infrared sensor - SGXV02-100 - FATRI France - thermopile / for portable infrared thermometer. https://www.directindustry.com/prod/fatri-france/product-234740-2362468.html.

30. Product finder encoders - Johannes Hübner Giessen. https://www.huebner-giessen.com/en/products/encoders/product-finder/?page=1.

31. Sensor Technology | Newark. https://mexico.newark.com/sensor-technology?ICID=I-CT-LP-TC-000023-SMART_IOT_PARKING-SENSING-APR21-W2467685.

32. InGaAs Photodiodes | OSI Optoelectronics. https://osioptoelectronics.com/standard-products/ingaas-photodiodes.aspx?gclid=CjwKCAjw5s6WBhA4EiwACGncZSMdJ3eolHSCfNKp-skPGY3aLxaq01qaOEa-ZasLEAQnRcVvkiB_ZhoCYmIQAvD_BwE.

33. SEMITEC KOREA – Thermistor,IR Sensor,Sensor assembly. http://eng.semiteckorea.com/.

34. Buy Obstacle Avoidance IR Sensor Module at Low Price In India. https://robu.in/product/ir-infrared-obstacle-avoidance-sensor-module/.

35. China Customized Infrared Sensor Manufacturers - Bulk Low Price Infrared Sensor in Stock - EPT. https://www.eptsensor.com/infrared-sensor/.

36. Infrared detectors | Hamamatsu Photonics. https://www.hamamatsu.com/eu/en/product/optical-sensors/infrared-detector.html.

37. Ultrasonic distance sensors | Baumer France. https://www.baumer.com/fr/en/product-overview/distance-measurement/ultrasonic-distance-sensors/c/290.

38. Mühendisler Elektrik San. ve Tic. Ltd. Şti. - SCAN. http://www.muhendisler.com.tr/content/blogsection/5/71/lang,turkish/index.html.

39. Buy Ultrasonic Sensors - Distrelec Germany. https://www.distrelec.de/en/sensors/ultrasonic-sensors/c/cat-DNAV_PL_1316.

40. Catalogs | Product Catalogues from Pepperl+Fuchs. https://www.pepperl-fuchs.com/global/en/17733.htm.

41. SENSORTEC Co., Ltd South Korea Manufacturers, suppliers. https://sensortec.tradekorea.com/main.do;JSESSIONID_TK=4f4MGUl7kpQkvkGK7p-bquaIeClygZycvh9575Wl777uUwMWHvII!-1458379923!2146405622.

42. Ultrasonic Sensor Module - Ultrasonic Motion Sensor Latest Price, Manufacturers & Suppliers. https://dir.indiamart.com/impcat/ultrasonic-sensor-module.html.

43. Solution by ultrasonic inspection・measurement product’s development | Japan Probe Co, Ltd. https://www.jp-probe.com/en/.

44. China Ultrasonic Sensor Manufacturers, Suppliers, Factory, Wholesale - ShenZhen OSENON Technology Co.,Ltd. https://www.osenon-ultrasonicsensors.com/ultrasonic-sensor-1.

45. JSLiDAR - HOME. http://jslidar.co.kr/en/.

46. Toshiba’s New Palm-sized Projector Unit Pushes LiDAR’s Detection Range to 300m, the World’s Longest, with Industry-leading Image Quality -Flexibility to combine multiple projectors allows customization of detection range and angle, realizes wide application from autonomous driving to infrastructure monitoring.- | Corporate Research & Development Center | Toshiba. https://www.global.toshiba/ww/technology/corporate/rdc/rd/topics/22/2203-02.html.

47. RoboSense LiDAR - Autonomous Driving, Robots, V2X. https://www.robosense.ai/en.

48. Top 10 LiDAR Manufacturers in World | Market Research Reports® Inc. https://www.marketresearchreports.com/blog/2019/04/22/top-10-lidar-manufacturers-world.

49. Samaras, C. & Meisterling, K. Life cycle assessment of greenhouse gas emissions from plug-in hybrid vehicles: Implications for policy. *Environ Sci Technol* **42**, 3170–3176 (2008).

50. BYD Qin EV | Specs | Dimensions | Range | Battery | Price | Qin Pro EV | WattEV2Buy. https://wattev2buy.com/electric-vehicles/byd-electric-vehicles/byd-qin-ev300-ev/.

51. Report: EV battery costs hit another low in 2021, but they might rise in 2022. https://www.greencarreports.com/news/1134307_report-ev-battery-costs-might-rise-in-2022.

52. ROBOSENSE RS-LiDAR-M1 is officially on sale priced at $1,898 - Geospatial World. https://www.geospatialworld.net/news/robosense-rs-lidar-m1-is-officially-on-sale-priced-at-1898/.

53. Meet WARLORD: Metawave Aims to Bring Millimeter-Wave RADAR Sensors to the Automotive Industry - News. https://www.allaboutcircuits.com/news/millimeter-wave-RADAR-sensors-entering-automotive-industry-Metawave/.

54. Compare electric vehicles - EV Database. https://ev-database.org/#sort:path~type~order=.rank~number~desc|range-slider-range:prev~next=0~1200|range-slider-acceleration:prev~next=2~23|range-slider-topspeed:prev~next=110~450|range-slider-battery:prev~next=10~200|range-slider-towweight:prev~next=0~2500|range-slider-fastcharge:prev~next=0~1500|paging:currentPage=0|paging:number=9.

55. Toyota BZ4X 2022 Price In Japan , Features And Specs - Ccarprice JPY. https://www.ccarprice.com/jp/toyota-bz4x-2022-price-in-japan-7627.

56. LS CH32 Automotive Grade 3D LiDAR designed for Automotive Grade Standards to meet the demands of L4 and L5 Autonomous Cars – Oz Robotics. https://ozrobotics.com/shop/ls-ch32-automotive-grade-3d-lidar/.

57. Amazon.co.jp: KIMISS REIWA 12V Universal Blind Spot Detection Monitoring System Kit BSD Sensor : Automotive. https://www.amazon.co.jp/-/en/KIMISS-Universal-Detection-Monitoring-System/dp/B08H81ZX9Y.

58. Hyundai Mobis MAR120 Blind Spot Radar (99140-AA020) Teardown. https://www.reverse-costing.com/teardowns/hyundai%20mobis_mar120_blind_spot_radar/#features.

59. Nissan LEAF gets upgrades for Japanese market. https://global.nissannews.com/en/releases/release-d47e2bd8192d14c383483ebde7066762-191216-01-e.

60. NISSAN LEAF EV | Specs | Range | Price | Battery | Charge Cost | WattEV2Buy. https://wattev2buy.com/electric-vehicles/nissan/nissan-leaf/.

61. LS CX16 3D Surround LiDAR for Autonomous Vehicle, ADAS, Smart Transportation, Service Robots, Logistics, Surveying, Mapping and Security – Oz Robotics. https://ozrobotics.com/shop/ls-cx16-3d-surround-lidar/.

62. Amazon.co.jp: Blind Spot Sensor 24GHZ Blind Spot Detection System Spot Detector [4 in 1] IP67 BSD, BSM, RCTA 12V Car Security Driving Alarm, Detects Vehicles From Rear : Automotive. https://www.amazon.co.jp/-/en/Detection-Detector-Security-Driving-Vehicles/dp/B081ND2HKQ/ref=pb_allspark_dp_sims_pao_desktop_session_based_2/356-6470956-1804326?pd_rd_w=y12Rm&pf_rd_p=2562f525-4075-4561-9863-48d7a49161bd&pf_rd_r=H1534SDT8TNY07SW53WH&pd_rd_r=2a8ac42a-3c09-43fa-92a0-74c62ae063ff&pd_rd_wg=nfgmO&pd_rd_i=B081ND2HKQ&psc=1.

63. Continental ARS5-A ARS540 4D Long-Range Radar (66 31 5 A55 CB1) Teardown. https://www.reverse-costing.com/teardowns/continental_ars5a_ars540_4d_longrange_radar/#features.

64. Altima Configurator | Packages | Nissan USA. https://www.nissanusa.com/shopping-tools/build-price/cars/altima/2022/fwd/29413:BABc0:AxZCUxWA/packages.

65. Tata Nexon EV XZ Plus Lux Dark Edition On Road Price (Electric(Battery)), Features & Specs, Images. https://www.cardekho.com/overview/Tata_Nexon_EV/Tata_Nexon_EV_XZ_Plus_Lux_Dark_Edition.htm.

66. OUSTER ANNOUNCES FIRST HIGH-PERFORMANCE TRUE SOLID-STATE DIGITAL LIDAR SENSOR. https://iot-automotive.news/ouster-announces-first-high-performance-true-solid-state-digital-lidar-sensor/.

67. Price - IONIQ 5 – HYDROGEN/ELECTRIC | HYUNDAI Motors. https://www.hyundai.com/kr/en/fcev-ev/ioniq5/price.

68. OLEI LR-16F 3D 16-channel LiDAR. https://morpheustek.com/shop/lidar/olei-3d-lidar/.

69. T-79: 79 GHz Radar Automotive Safety Radar | Ainstein. https://ainstein.ai/vehicle-radar/short-range-wideband-high-resolution-automotive-radar-sensor/.

70. Costs and Outlook of On-Board Equipment for Connected Vehicles | U.S. Department of Transportation. https://www.itskrs.its.dot.gov/its/benecost.nsf/ID/26d0d89dc2f1144185257bb40064d1b6.

71. Tesla Motors Inc. Is Using NVIDIA Corporation’s Drive PX 2 | The Motley Fool. https://www.fool.com/investing/2016/10/27/tesla-motors-inc-is-using-nvidia-corporations-driv.aspx.

72. Amazon.com: Garmin Drive 51 USA LM GPS Navigator System with Lifetime Maps, Spoken Turn-By-Turn Directions, Direct Access, Driver Alerts, TripAdvisor and Foursquare Data : Electronics. https://www.amazon.com/Garmin-Navigator-Directions-TripAdvisor-Foursquare/dp/B01NBM6JUV/ref=sr_1_3?c=ts&keywords=Vehicle%2BGPS%2BUnits%2B%26%2BEquipment&qid=1649080396&s=gps&sr=1-3&ts_id=559938&th=1.

73. Cellular-V2X (C-V2X) communication modules that use wireless wide-area network (WWAN) cellular modems to broadcast and receive basic safety messages (BSM) can be supplied to automobile manufacturers for less than $222 per unit. | U.S. Department of Transportation. https://www.itskrs.its.dot.gov/its/benecost.nsf/ID/8c2875e02cd554d3852582910068e4f8.

74. 2021 Ford® Mustang Mach-E SUV | All-Electric & Exhilarating. https://www.ford.com/suvs/mach-e/2021/.

75. Ford Mustang Mach-E ER RWD - (Battery Energy 98.7 kWh, Torque 430 Nm) - Electric Vehicles Database. https://evehiclesdb.com/transport/ford-mustang-mach-e-er-rwd/.

76. Luminar Surges On Plan To Supply Laser Sensors For Nvidia’s Self-Driving Car Platform. https://www.forbes.com/sites/alanohnsman/2021/11/09/luminar-to-supply-laser-sensors-for-nvidias-self-driving-car-platform/?sh=531d4d245645.

77. Ford Co-Pilot360™ Technology Adds Hands-Free Driving, Over-The-Air Updates and More to Help Ford Customers Feel More Relaxed and Confident | Ford Media Center. https://media.ford.com/content/fordmedia/fna/us/en/news/2020/06/18/ford-co-pilot360-technology-adds-hands-free-driving.html.

78. AR1820HSSC12SHQAH3-GEVB onsemi | Mouser. https://www.mouser.com/ProductDetail/onsemi/AR1820HSSC12SHQAH3-GEVB?qs=GWuLPj4sST14IGqDQio0oA%3D%3D.

79. onsemi AR1820HS CMOS Image Sensor | EBV Elektronik. https://www.avnet.com/wps/portal/ebv/products/new-products/npi/2017/onsemi-AR1820HS.

80. AR1820HS: CMOS Image Sensor, 18 MP, 1/2.3". https://www.onsemi.com/products/sensors/image-sensors/ar1820hs.

81. Build & Price | Ford® Mach-E All-Electric SUV. https://www.ford.com/buy/mach-e/build-and-price.html?config=WANAB-CGW-2022-CX727#/extras/AACVB?series=VS-KX&powertrain=DR--F_EN-C2_HTHAE_TR-WA&paint=PNZAT&features=HTSADincluded.

82. Design Your Model S | Tesla. https://www.tesla.com/models/design#overview.

83. New Velodyne LiDAR Puck VLP-16 - SALE !! https://www.nusahendheld3d.com/3d-lidar-sensors/new-velodyne-lidar-puck-vlp-16---sale.

84. Amazon.com: CarBest Ultrasonic Blind Spot Detection System BSD Change Lane Safer BSA BSM Blind Spot Monitoring Assistant Car Driving Security with Reverse Parking System : Electronics. https://www.amazon.com/CarBest-Ultrasonic-Detection-Monitoring-Assistant/dp/B07HTNCTGG/ref=sr_1_11?crid=263IU9IHFRWFL&keywords=ultrasonic+sensor+car&qid=1645263980&sprefix=ultrasonic+sensor+car%2Caps%2C401&sr=8-11.

85. 2017 Zacua MX2 18 kWh (46 Hp) Electric | Technical specs, data, fuel consumption, Dimensions. https://www.auto-data.net/en/zacua-mx2-18-kwh-46hp-electric-38524.

86. There's a 100% Electric Car built by Mexican women, its name is Zacua – The Yucatan Times. https://www.theyucatantimes.com/2022/02/theres-a-100-electric-car-built-by-mexican-women-its-name-is-zacua/.

87. 2017 Zacua MX2 18 kWh (46 Hp) Electric | Technical specs, data, fuel consumption, Dimensions. https://www.auto-data.net/en/zacua-mx2-18-kwh-46hp-electric-38524.

88. Horizon lidar sensor - Livox. https://www.livoxtech.com/horizon.

89. Konfigurator. https://www.volkswagen.de/de/konfigurator.html/__app/%2Fid-4.app.

90. New - Sick 3D LiDAR sensors LD-MRS/LD-MRS 8-Layer/Outdoor - SALE !! https://www.nusahendheld3d.com/3d-lidar-sensors/new---sick-3d-lidar-sensors-ld-mrsld-mrs-8-layeroutdoor---sale.

91. PoeHXtyy Ultrasonic Blind Spot Detection System BSD Lane Change Safe Assistant for Monitoring Blind Spot Safety Driving : Amazon.de: Automotive. https://www.amazon.de/-/en/PoeHXtyy-Ultrasonic-Detection-Assistant-Monitoring/dp/B085VPF6ZY/ref=pd_lpo_2?pd_rd_i=B085VPF6ZY&psc=1.

92. le Gac, D. COMPLETE TEARDOWN WITH: • Detailed photos Bosch LRR4 77GHz Long Range Radar Sensor Title: Bosch LLR4 77GHz Radar The fourth generation of Bosch long range radar sensor sets new standards for a more elegant, compact and cost effective module. (2017).

93. Konfigurator. https://configure.bmw.de/de_DE/configure/G26E/71AW/FKFSW,P0668,S01CB,S0230,S0255,S02PA,S02VB,S03F0,S0428,S0431,S0493,S04AT,S04AW,S04U9,S0534,S0544,S0548,S05AQ,S05DA,S05DM,S0654,S06AE,S06AF,S06AK,S06C4,S06U2,S06UX,S06VB,S06WC,S0801,S0851,S0879,S08R9,S08TF,S09QX/EI0000E6,SE000001.

94. What is night vision, how does it work, and do I really need it in my next car? - ExtremeTech. https://www.extremetech.com/extreme/193402-what-is-night-vision-how-does-it-work-and-do-i-really-need-it-in-my-next-car.

95. BOSCH LRR4 Long Range Radar (4N0 907 561) Teardown. https://www.reverse-costing.com/teardowns/bosch_lrr4/#features.

96. Build Your Own – Add Packages, Features, Options – BMW USA. https://www.bmwusa.com/build-your-own.iframe.html#/studio/ep1ll293/options.

97. Yerli otomobil rakiplerine teknik fark atacak. https://www.haberturk.com/yerli-otomobil-rakiplerine-teknik-fark-atacak-2553792-ekonomi.

98. How Much Will TOGG Cost? What is the Sales Price of the Domestic Car? https://www.raillynews.com/2021/11/how-much-will-the-togg-cost-be-how-much-is-the-sales-price-of-the-domestic-car/.

99. New- Quanergy M8 Prime Plus 3D Lidar Sensors - SALE !! https://www.nusahendheld3d.com/new--quanergy-m8-prime-plus-3d-lidar-sensors---sale.

100. Configurateur Renault ZOE – Renault. https://www.renault.fr/vehicules-electriques/zoe/configurateur.html.

101. Cepton sets sub-$1000 price for latest auto lidar product. https://optics.org/news/11/9/40.

102. Valeo Retrofit Kit Blind Spot Assistant 632300 : Amazon.de: Electronics & Photo. https://www.amazon.de/-/en/Valeo-Retrofit-Blind-Assistant-632300/dp/B084BKQ14V/ref=pd_lpo_1?pd_rd_i=B084BKQ14V&psc=1.

103. smartmicro Radar Sensor: UMRR-96 | Level Five Supplies. https://levelfivesupplies.com/product/automotive-radar-sensor-umrr-96-type-153/.

104. Qatar General Electricity & water Corporation - المؤسسة العامة القطرية للكهرباء والماء. https://www.km.qa/Pages/default.aspx.

105. Kavas-Torris, O., Cantas, M. R., Aksun-Guvenc, B., Guvenc, L. & Cime, K. M. The Effects of Varying Penetration Rates of L4-L5 Autonomous Vehicles on Fuel Efficiency and Mobility of Traffic Networks ADAS enhanced Powertrain Controls View project The Effects of Varying Penetration Rates of L4-L5 Autonomous Vehicles on Fuel Efficiency and Mobility of Traffic Networks. doi:10.4271/2020-01-0137.

106. Recycling and Climate Change | NC DEQ. https://deq.nc.gov/conservation/recycling/recycling-climate-change.

107. Sixth Assessment Report — IPCC. https://www.ipcc.ch/assessment-report/ar6/.

108. McKone, T. E. *et al.* Grand challenges for life-cycle assessment of biofuels. *Environ Sci Technol* **45**, 1751–1756 (2011).

109. Lloyd, S. M. & Ries, R. Characterizing, Propagating, and Analyzing Uncertainty in Life-Cycle Assessment: A Survey of Quantitative Approaches. *J Ind Ecol* **11**, 161–179 (2007).
