# Supplementary material for: Rebound effects undermine carbon footprint reduction potential of autonomous electric vehicles
Source: Nat Commun. 2023 Oct 6;14:6258. doi: 10.1038/s41467-023-41992-2 (PMC10558530; doi:10.1038/s41467-023-41992-2)
Supplement: Supplementary file 3 — Reporting Summary [file 41467_2023_41992_MOESM3_ESM.pdf]

Corresponding author(s): Nuri C. Onat
Last updated by author(s): September 09, 2023

Reporting Summary

Nature Portfolio wishes to improve the reproducibility of the work that we publish. This form provides structure for consistency and transparency in reporting. For further information on Nature Portfolio policies, see our Editorial Policies and the Editorial Policy Checklist.

Statistics

For all statistical analyses, confirm that the following items are present in the figure legend, table legend, main text, or Methods section.

n/a	Confirmed	
☐	☒	The exact sample size (*n*) for each experimental group/condition, given as a discrete number and unit of measurement
☐	☒	A statement on whether measurements were taken from distinct samples or whether the same sample was measured repeatedly
☒	☐	The statistical test(s) used AND whether they are one- or two-sided<br>*Only common tests should be described solely by name; describe more complex techniques in the Methods section.*
☐	☒	A description of all covariates tested
☐	☒	A description of any assumptions or corrections, such as tests of normality and adjustment for multiple comparisons
☐	☒	A full description of the statistical parameters including central tendency (e.g. means) or other basic estimates (e.g. regression coefficient) AND variation (e.g. standard deviation) or associated estimates of uncertainty (e.g. confidence intervals)
☒	☐	For null hypothesis testing, the test statistic (e.g. *F*, *t*, *r*) with confidence intervals, effect sizes, degrees of freedom and *P* value noted<br>*Give P values as exact values whenever suitable.*
☐	☒	For Bayesian analysis, information on the choice of priors and Markov chain Monte Carlo settings
☒	☐	For hierarchical and complex designs, identification of the appropriate level for tests and full reporting of outcomes
☒	☐	Estimates of effect sizes (e.g. Cohen's *d*, Pearson's *r*), indicating how they were calculated

Our web collection on statistics for biologists contains articles on many of the points above.

Software and code

Policy information about availability of computer code

Data collection	There are several sources of data: Survey (primery data), MRIO EXIOBASE database 3.8.2 (secondary data), and life cycle inventory and cost estimations from publicly available sources such as EIA, National Statistics, GREET 2 (2022) model for material composition of vehicles, and some manufacturer's data. For surveying, we used Qualtrics online surveying platform.
Data analysis	We use Pycharm as Python IDE coding software (version 2020.2.3) , MS excel for calculations (Microsoft Office 365 2021). For data visualization, we used Vensim version 9.3.2 (only visualization of the CLD), Adobe Photoshop CS6 (version 2012), Autodesk Sketchbook 7 (2014 version), and Microsoft Power point (Microsoft Office 365 2021).

For manuscripts utilizing custom algorithms or software that are central to the research but not yet described in published literature, software must be made available to editors and reviewers. We strongly encourage code deposition in a community repository (e.g. GitHub). See the Nature Portfolio guidelines for submitting code & software for further information.

Data

Policy information about availability of data

All manuscripts must include a data availability statement. This statement should provide the following information, where applicable:

- Accession codes, unique identifiers, or web links for publicly available datasets
- A description of any restrictions on data availability
- For clinical datasets or third party data, please ensure that the statement adheres to our policy

All data and codes are provided as a link in the Supplementary information file.

Research involving human participants, their data, or biological material

Policy information about studies with human participants or human data. See also policy information about sex, gender (identity/presentation), and sexual orientation and race, ethnicity and racism.

Reporting on sex and gender	Sex and gender were not considered in this study.
Reporting on race, ethnicity, or other socially relevant groupings	We didn't consider race, ethnicity. We included self-reported income, marital status, employment, education level of respondents in our analysis.
Population characteristics	Survey was designed for this paper as a web-based survey questionnaire was developed which comprises of collecting respondents' socio-economic information (such as age, education, employment status, driving experience, and their belief and attitudes towards AVs technology). The covariate-relevant characteristics are presented in Supplementary Table 5.
Recruitment	The questionnaire was designed in Arabic and English languages using the Qualtrics platform, which is a web-based survey tool. The questionnaire comprised three different sections. The first section includes questions regarding socio-demographic characteristics of the respondents such as age, ethnicity, average income, educational status, occupational status. The second section included some questions related to commuting behaviors. In the third section, questions related to individuals' knowledge about AV, perceptions of AV's safety, performance in harsh environmental conditions, security, travel time, congestions, comfort, and operational costs, and their preference of shifting to AV. A brief list of the questions and the possible answer choices are provided in the SI file in Section 2. Most of the questions in the third section were not utilized by this study as it is not included in our scope to assess people's perception of AVs in terms of what they think about their benefits, drawbacks, and possibility of switching to AVs when they become available. Efforts have been made to reduce the self selection bias as much as possible by offering an incentive of 100 QAR, for which the winners are chosen randomly. Rewards can assist people with moderate opinions overcome their lack of willingness (Marinescu et al., 2018)
Ethics oversight	An approval was obtained from Qatar University's ethical committee (QU-IRB) before distributing the questionnaires. The respondents approved a consent before they proceed to the Survey questions. The full consent statement is shared in the Supplementary information file. Participants consent statement was added to the surveying section in the manuscript.

Note that full information on the approval of the study protocol must also be provided in the manuscript.

Field-specific reporting

Please select the one below that is the best fit for your research. If you are not sure, read the appropriate sections before making your selection.

☐ Life sciences ☒ Behavioural & social sciences ☐ Ecological, evolutionary & environmental sciences

For a reference copy of the document with all sections, see nature.com/documents/nr-reporting-summary-flat.pdf

Behavioural & social sciences study design

All studies must disclose on these points even when the disclosure is negative.

Study description	The study is a stated preference survey designed to understand Qatari residents' preferences among autonomous vehicles. The data collected is primarily quantitative, as it involves respondents indicating their choices based on perceptions of autonomous vehicles presented in the survey. Demographic data was collected from the respondents including age, marital status, employment type, marital status, education level, income, number of adults in household, and number of cars in household. Other variables were collected including travelling behaviors variables such as number of years of driving experience, car owning, average travelled distances per year, and willingness to pay for a new car. Variables related to perceptions about autonomous vehicles were also collected including background knowledge of AVs, perceptions about proposed benefits including reduced congestion, fuel consumption, and travel time, proposed drawbacks including accidents between AVs and non-autonomous cars, hackers' threats, and increased maintenance operations.
Research sample	The research sample consists of Qatari residents. The original sample includes 589 respondents who were residing in the state of

Research sample	Qatar. The sample must only include adults of 18 years old or older as the main aim of the survey is to investigate how autonomous vehicles would affect annual driving distances. The survey is not restricted to any of the demographic variables listed before as it could include ,for example, high school, diploma, bachelor's or graduate degrees holders, this is also the same case for all other variables. The population we want to investigate includes only light-duty vehicle licensed drivers. For the period 2018-2022, about 765,600 licenses of this category were either issued or renewed (we took the five years period as driving licenses in Qatar are valid for only five years). This estimation included building an Exponential Triple Smoothing forecasting model to forecast the number of renewal and issuance of licenses in 2021 and 2022 as the official published data is limited to 2020. We calculate the required sample size using the following formula:<br>N = Z(α/2)2 *p*(1-p) / MOE2,<br><br>• Zα/2: the critical value of the Normal distribution at α/2 (at the current confidence level of 90%, α is 0.1 and the critical value is 1.645)<br>• N: Sample size<br>• MOE: the margin of error (5% assumed value)<br>• P: the sample proportion (50% assumed as recommended by default)<br>• N: the population size (765600)<br><br>Using this formula, we get the required sample size to be 271. Therefore, the sample size of 330 (after screening) is representative of the population we aim to investigate since it is larger than the minimum required sample size.
Sampling strategy	The sampling procedure was random as the survey was distributed through various forums on social media such as Facebook, Instagram, and other platforms, as well as official channels within Qatar University where researchers collected information from respondents in-person. The same procedure was also followed outside the university campus where the researcher's collected information from respondents in public place within Doha including Katara. he population we want to investigate includes only light-duty vehicle licensed drivers. For the period 2018-2022, about 765,600 licenses of this category were either issued or renewed (we took the five years period as driving licenses in Qatar are valid for only five years). This estimation included building an Exponential Triple Smoothing forecasting model to forecast the number of renewal and issuance of licenses in 2021 and 2022 as the official published data is limited to 2020. We calculate the required sample size using the following formula:<br>N = Z(α/2)2 *p*(1-p) / MOE2,<br><br>• Zα/2: the critical value of the Normal distribution at α/2 (at the current confidence level of 90%, α is 0.1 and the critical value is 1.645)<br>• N: Sample size<br>• MOE: the margin of error (5% assumed value)<br>• P: the sample proportion (50% assumed as recommended by default)<br>• N: the population size (765600)
Data collection	The data was collected through a web-based survey questionnaire developed using the Qualtrics platform. In addition, the researchers collected information within Qatar University by using tablets. Only the researchers and respondents were present at the time of collection. The researcher is considered to be blinded to the experimental condition as, In this study, we used third-party survey platform (Qualtrics) that allow for controlled data collection and anonymization. This ensures that the researcher does not have access to individual responses during the data collection process. As for blindness to study hypothesis, the researcher is blinded as collaborative survey construction took place where more than one researcher each handled a different task within survey design including survey construction, data collection, and data analysis.
Timing	The timeline of data collection starts from 6th January 2019 to 12th December 2019.
Data exclusions	The data was filtered for the respondents of age at least 18 years (legal age limit qualifying for driving license), missing income data, missing rebound-effect related question data, and missing data related to AV background knowledge. In this regard, 9, 145, 104, 1 respondents were eliminated from the sample, respectively. Thus, 330 respondents were considered as a final sample for the analyses.
Non-participation	None of the participants opted to drop out of their participation.
Randomization	No experimental groups were created; thus, no randomization took place.

Reporting for specific materials, systems and methods

We require information from authors about some types of materials, experimental systems and methods used in many studies. Here, indicate whether each material, system or method listed is relevant to your study. If you are not sure if a list item applies to your research, read the appropriate section before selecting a response.

Materials & experimental systems

n/a	Involved in the study
☒	☐ Antibodies
☒	☐ Eukaryotic cell lines
☒	☐ Palaeontology and archaeology
☒	☐ Animals and other organisms
☒	☐ Clinical data
☒	☐ Dual use research of concern
☒	☐ Plants

Methods

n/a	Involved in the study
☒	☐ ChIP-seq
☒	☐ Flow cytometry
☒	☐ MRI-based neuroimaging
